# Design, synthesis, and biological evaluation of novel *N*^*4*^-substituted sulfonamides: acetamides derivatives as dihydrofolate reductase (DHFR) inhibitors

**DOI:** 10.1186/s13065-019-0603-x

**Published:** 2019-07-11

**Authors:** Essam M. Hussein, Munirah M. Al-Rooqi, Shimaa M. Abd El-Galil, Saleh A. Ahmed

**Affiliations:** 1 Department of Chemistry, Faculty of Applied Science, Umm Al-Qura University, Makkah, 21955 Saudi Arabia; 2 Chemistry Department, Faculty of Science, Assiut University, Assiut, 71516 Egypt; 3 Department of Pharmaceutical Organic Chemistry, Faculty of Pharmacy (Girls), Al-azhar University, Nacr City, Cairo, Egypt

**Keywords:** Sulfonamide, Anticancer, Antimicrobial, Acetamides, Molecular docking, Structure–activity relationship (SAR), DHFR inhibitors

## Abstract

**Background:**

Sulfonamide derivatives are of great attention due to their wide spectrum of biological activities. Sulfonamides conjugated with acetamide fragments exhibit antimicrobial and anticancer activities. The inhibition dihydrofolate reductase (DHFR) is considered as one of the most prominent mechanism though which sulfonamide derivatives exhibits antimicrobial and antitumor activities.

**Results:**

In this study, a new series of 2-(arylamino)acetamides and *N*-arylacetamides containing sulfonamide moieties were designed, synthesized, characterized and assessed for their antimicrobial activity and screened for cytotoxic activity against human lung carcinoma (A-549) and human breast carcinoma (MCF-7) cell lines. A molecular docking study was performed to identify the mode of action of the synthesized compounds and their good binding interactions were observed with the active sites of dihydrofolate reductase (DHFR).

**Conclusion:**

Most of the synthesized compounds showed significant activity against A-549 and MCF-7 when compared to 5-Fluorouracil (5-FU), which was used as a reference drug. Some of these synthesized compounds are active as antibacterial and antifungal agents.
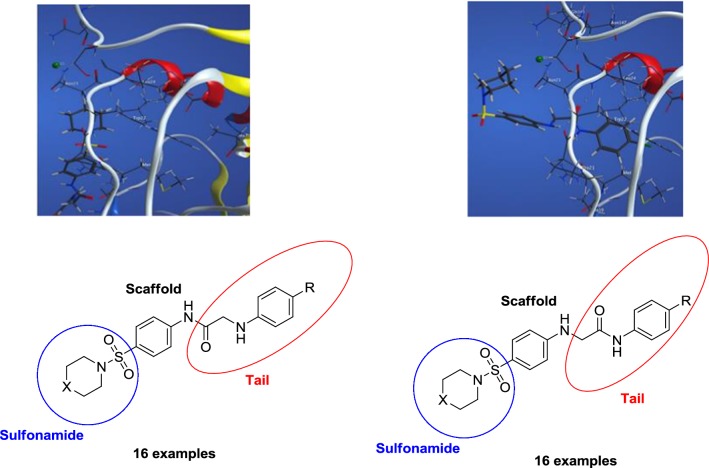

**Electronic supplementary material:**

The online version of this article (10.1186/s13065-019-0603-x) contains supplementary material, which is available to authorized users.

## Introduction

Sulfonamides have attracted considerable deal of interest over past decades due to their broad and wide spectrum of biological activities which includes antibacterial [1], antifungal [2], hypoglycemic [3], anti-thyroid [4], diuretic [5, 6] and anti-HIV properties [7]. Recently, a large number of structurally novel sulfonamides have been reported to show substantial in vitro and in vivo antitumor activity [8–15]. The anticancer activity is exerted by the sulfonamides through a wide range of mechanisms, such as cell cycle arrest in the G1 phase [16], inhibition of carbonic anhydrase (CA) [17], matrix metalloproteinase (MMPs) [18], NADH oxidase [19], cyclin-dependent kinase (CDK) [20], methionine aminopeptidases (MetAPs) [21], histone deacetylases (HDACs) [22], binding to β-Tubulin, and disruption of microtubule assembly [23].

On other hand, compounds with acetamide linkage exhibit variety of applications, which are well documented. The Lewis acid property of acetamides renders them useful as analytical reagents and in the preparation of a number of coordination complexes [24]. The acetamide functional group is responsible for antimicrobial [25, 26], antioxidant [27, 28], narcolepsy treatment [29], anti-inflammatory [30, 31], platelet aggregation inhibitory [32], and urease inhibitory activities [33]. The acetamides and their analogues are also well studied as chemotherapeutic agents [34, 35].

Dihydrofolate reductase (DHFR) is a key enzyme that catalyzes the NADPH-dependent reduction of 7,8-dihydrofolate (DHF) to 5,6,7,8-tetrahydrofolate (THF): DHF + NADPH + H^+^ → THF + NADP^+^, which is the precursor of the co-factors required for the biosynthesis of purine nucleotides, thymidine (precursor for DNA replication) and several amino acids [36]. Thus, inhibition of DHFR can lead to the disruption of DNA synthesis and the death of the rapidly proliferating cells [36, 37]. In addition to this, bacteria also need DHFR to grow and multiply and hence inhibitors selective for bacterial against host DHFR have found application as antibacterial agents [38]. These two important aspects render DHFR enzyme as a key target for both antimicrobial and antitumor drug design.

The sulfonamide group conjugated with acetamides possessing different aryl, heteroaryl as well as alkyl substituents exhibits immense pharmacological potential, particularly sulfonamides containing short amine fragments exhibits encouraging anticancer activity [39–41]. This accounts for the growing interest in the synthesis, biological properties and structure activity relationships of sulfonamide-acetamide derivatives.

Based on these prior observations relating to the synthesis of sulfonamide derivatives [42, 43] and bioactive nitrogen-containing heterocyclic agents [44–48] we envisaged that sulfonamides bearing acetamide pharmacophores could be very efficient for antimicrobial and anticancer activity. In the present work, we report the synthesis of some novel sulfonamide-*N*^*4*^-acetamide derivatives and their evaluation of their cytotoxic activity against human lung carcinoma (A-549) and human breast carcinoma (MCF-7) cell lines.

## Results and discussion

### Chemistry

The present study deals with the design and synthesis of some acetamide derivatives having different aryl substituents (tails) conjugated with biologically active sulfonamide moiety in order to explore their combined effect on the antimicrobial and antitumor activities, and study their structure–activity relationship (SAR).

**Fig. 1:**
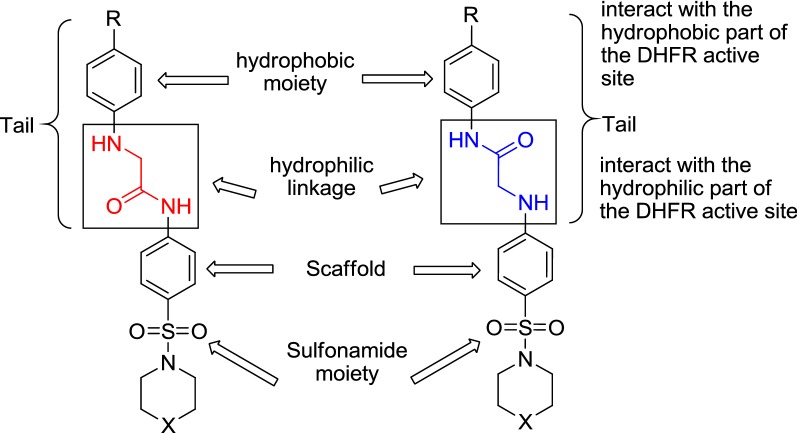
Structural elements of DHFR inhibitors in the DHFR enzymatic active site

As the DHFR inhibition is considered as one of the most prominent mechanism for the antimicrobial and antitumor activities [49–51], the synthesized compounds were intended to comply with the pharmacophore present in compounds that may act as DHFR inhibitors. The sulfonamide is attached to a scaffold, which is frequently a benzene ring, and a tail comprising of groups such as 2-(arylamino)acetamide or *N*-arylacetamide is attached to scaffold. The tail possesses a hydrophobic moiety, which is able to interact with the hydrophobic part of the active site and a hydrophilic linker which can interact with the hydrophilic part of the DHFR active site (Fig. 1). This pharmacophore was designed from the analysis of the DHFRs active site and from the structure of inhibitors were described in literature [52, 53].

In this work, the starting key materials 4-(piperidin-1-ylsulfonyl)aniline (**1a**) and 4-(morpholin-4-ylsulfonyl)aniline (**1b**) were prepared accordingly as the reported method [42], and were converted to the corresponding chloroacetamide derivatives **2a**,**b** in excellent yields (92–95%) by reaction with chloroacetyl chloride in DMF at room temperature.

**Scheme 1:**
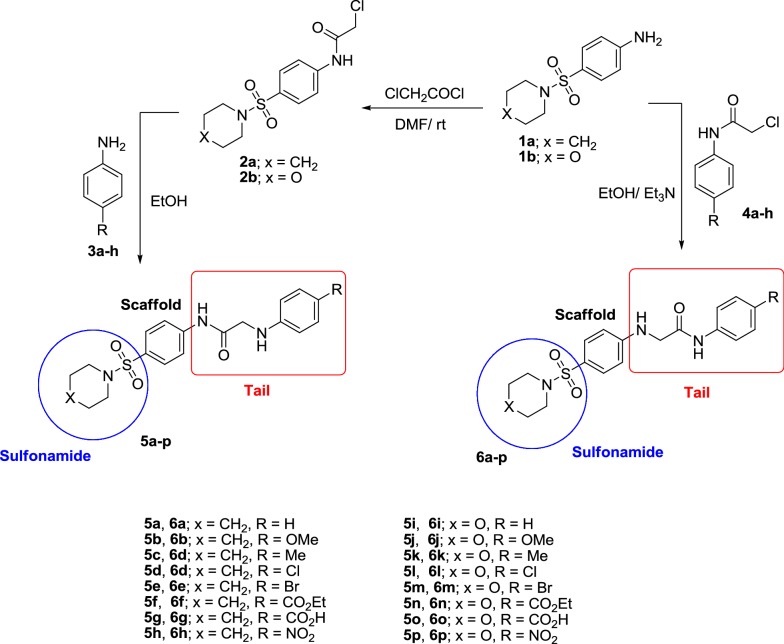
Synthesis of novel *N*^*4*^-substituted sulfonamide derivatives **5a**–**p** and **6a**–**p**

The target compounds 2-(arylamino)-*N*-(4-(piperidin-1-ylsulfonyl)phenyl)acetamides **5a**–**h** and 2-(arylamino)-*N*-(4-(morpholino-sulfonyl)phenyl)acetamides **5i**–**p**, were obtained in moderate to good yields (51–84%) by refluxing chloroacetamide derivatives **2a** and **2b**, respectively, with arylamines (namely; aniline, 4-methoxyaniline, 4-methylaniline, 4-chloroaniline, 4-bromoaniline, ethyl 4-aminobenzoate, 4-aminobenzoic acid, and 4-nitroaniline) in absolute ethanol for 3–5 h. (Scheme 1).

On the other hand, 2-chloro-*N*-arylacetamides **4a**–**h** were easily prepared by the reaction of arylamines (namely; aniline, 4-methoxyaniline, 4-methylaniline, 4-chloroaniline, 4-bromoaniline, ethyl 4-aminobenzoate, 4-aminobenzoic acid, and 4-nitroaniline) with chloroacetyl chloride in DMF at room temperature.

Reaction of 2-chloro-*N*-arylacetamides **4a**–**h** with sulfonamide derivatives **1a** and **1b** in ethanol under refluxing conditions afforded the target compounds *N*-aryl-2-(4-(piperidin-1-ylsulfonyl)phenylamino)-acetamides **6a**–**h** and *N*-aryl-2-(4-(morpholinosulfonyl)-phenylamino)-acetamides **6i**–**p**, respectively, in good to excellent yields (57–97%) (Scheme 1). The reactions were performed in the presence of catalytic amount of triethylamine as a basic catalyst with a reaction time of 4-6 h. The structures of all synthesized compounds **2a**,**b**, **5a**–**p** and **6a**–**p** were well-established on the basis of FT-IR, ^1^H-NMR, ^13^C-NMR, and DEPT-135 data (c.f. “Experimental” section). The FT-IR spectra of compounds **5a**–**p** displayed the presence of characteristic absorption bands at 3499–3330 and 3365–3191 cm^−1^ for two NH groups, 1722–1680 cm^−1^ for (C=O) groups. Furthermore, to entirely confirm the chemical structures of the products, intensive 1D (^1^H, ^13^C, and DEPT-135) NMR were conducted in DMSO-*d*_*6*_. For example, analysis of the ^13^C and ^13^C-DEPT-135 NMR spectra of **5h** indicated the presence of 13 signals (4 aromatic CH’s, 4 aromatic quaternary carbons, 4 methylene carbons, and one carbonyl carbon). Its ^1^H-NMR spectrum showed two downfield singlet signals at 10.75 and 10.10 ppm for two NH protons. Two doublets at 7.94 and 6.60 ppm (*J* = 11.0 Hz) for the protons of 4-nitrophenyl moiety and two doublets at 7.84 and 7.70 ppm (*J* = 10.5 Hz) for aromatic CH’s protons of the scaffold moiety were present. In addition, a singlet signal at 4.32 ppm for the tail methylene protons and three multiplets at 2.85–2.83, 1.52–1.51, and 1.34–1.33 ppm for the piperidinyl ring protons were recorded.

On the other hand, the FT-IR spectra of compounds **6a**–**p**, showed the presence of characteristic absorption bands at 3444–3275 and 3365–3203 cm^−1^ for two NH groups, 1721–1641 cm^−1^ for (C=O) groups. As a representative example, the ^13^C and ^13^C-DEPT-135 NMR spectra of **6p** showed the presence of 12 signals (4 aromatic CH’s, 4 aromatic quaternary carbons, 3 methylene carbons, and one carbonyl carbon). Its ^1^H-NMR spectrum exhibited two singlet signals at 10.94 and 6.11 ppm for two NH protons. Two doublets at 8.24 and 6.87 ppm (*J* = 7.5 Hz) for the protons of 4-nitrophenyl moiety and two doublets at 7.84 and 7.35 ppm (*J* = 8.0 Hz) for aromatic CH’s protons of the scaffold moiety were present. In addition, a singlet signal at 4.35 ppm for the tail methylene protons and two multiplets at 3.60 and 2.78 ppm for the morpholinyl ring protons were recorded.

### Antimicrobial activity

The novel compounds were evaluated for antimicrobial activity against two strains of Gram-positive bacteria known as *S. aureus* (RCMB010010), *and B. subtilis* RCMB 015 (1) NRRL B-543, as well as two strains of Gram-negative bacteria namely *E. coli* (RCMB 010052) ATCC 25955, and *P. vulgaris* RCMB 004 (1) ATCC 13315, in addition to two types of fungi namely *A. fumigatus* (RCMB 002008), and *C. albicans* RCMB 005003 (1) ATCC 10231.

**Fig. 2:**
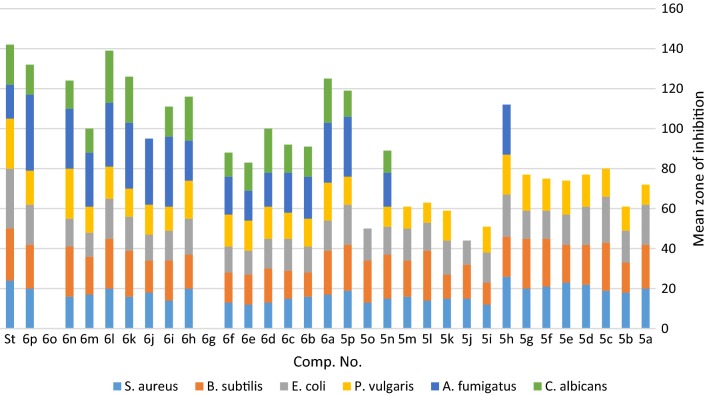
Comparison of the antimicrobial activity of the newly synthesized compounds

The result of the antimicrobial assay of the synthesized compounds is given in Table 1 and Fig. 2. It is observed that some of the compounds showed higher antimicrobial activity compared to the reference drugs. These compounds have given the best results in the inhibition of different types of bacteria and fungi; compound **5h** against the *S. aureus*, the zone of inhibition with ZOI value 26, compounds **5g**, **5l**, **6l** and **6n** against *B. Subtili* having ZOI value 25, compound **5c** against *E. coli* with ZOI value 23, while, compound **6n** against *P. vulgaris* having ZOI value 25. Moreover, compounds **5h**, **5n**, **5p**, **6a**, **6b**, **6c**, **6d**, **6f**, **6h**, **6i**, **6j**, **6k**, **6l**, **6m**, **6n** and **6p** against *A. fumigatus*, having (ZOI) values 25, 17, 30, 30, 21, 20, 17, 19, 20, 35, 33, 33, 32, 27, 30, 38; respectively. Furthermore, the following compounds **6a**, **6d**, **6h** having ZOI value 22 while, compounds **6k** and **6l** having ZOI values 23, 26; respectively against *C. Albicans*. It is clearly evident from the antimicrobial results that the synthesized compounds **6l** and **6n** exhibit dual activities as promising antibacterial and antifungal agents.

**Table 1:** Antimicrobial activity of newly synthesized compounds

Comp. no.	Gram (+ve) bacteria		Gram (−ve) bacteria		Fungi	
	*S. aureus*	*B. subtilis*	*E. coli*	*P. vulgaris*	*A. fumigatus*	*C. albicans*
**5a**	20	22	20	10	–	–
**5b**	18	15	16	12	–	–
**5c**	19	24	23	14	–	–
**5d**	22	20	19	16	–	–
**5e**	23	19	15	17	–	–
**5f**	21	24	14	16	–	–
**5g**	20	25	14	18	–	–
**5h**	26	20	21	20	25	–
**5i**	12	11	15	13	–	–
**5j**	15	17	12	–	–	–
**5k**	15	12	17	15	–	–
**5l**	14	25	14	10	–	–
**5m**	16	18	16	11	–	–
**5n**	15	22	14	10	17	11
**5o**	13	21	16	–	–	–
**5p**	19	23	20	14	30	13
**6a**	17	22	15	19	30	22
**6b**	16	12	13	14	21	15
**6c**	15	14	16	13	20	14
**6d**	13	17	15	16	17	22
**6e**	12	15	12	15	15	14
**6f**	13	15	13	16	19	12
**6g**	–	–	–	–	–	–
**6h**	20	17	18	19	20	22
**6i**	14	20	15	12	35	15
**6j**	18	16	13	15	33	–
**6k**	16	23	17	14	33	23
**6l**	20	25	20	16	32	26
**6m**	17	19	12	13	27	12
**6n**	16	25	14	25	30	14
**6o**	–	–	–	–	–	–
**6p**	20	22	20	17	38	15
St^a^	24	26	30	25	17	20

Mean zone of inhibition in mm ± standard deviation (S.D.)

^—^ No activity

^a^Reference controls for the microorganisms are “*Gentamycin*” (for the Gram +ve and Gram −ve bacteria), and “*Ketoconazol*” for Fungi

From all the previous data, its be concluded that, the following compounds **5g, 5h, 5l, 6l** and **6n** are the highly active compounds which have antibacterial activity against strains of Gram-positive bacteria. While, compounds **5c** and **6n** showing activity against strains of Gram-negative bacteria. All compared to Gentamycin as antibacterial reference drug. Moreover, compounds **5h, 5n, 5p, 6a, 6b, 6c, 6d**, **6f**, **6h**, **6i**, **6j**, **6k**, **6l**, **6m**, **6n**, **6p** acted as antifungal agents compared to Ketoconazole as a reference drug.

### In vitro anticancer activity

All new tested compounds were screened against human lung carcinoma (A-549) and breast carcinoma (MCF-7). The final result of evaluation were expressed as IC_50_ (the required concentration which can inhibit 50% of cancer cells viability). Results are explained in Tables 2 and 3. The reference control was 5-Fluorouracil (5-FU).

**Table 2:** *In vitro* anticancer screening of the synthesized compounds against human lung carcinoma cell line (A-549)

Comp. no.	Validity for sample conc.											IC_50_ (μg mL^−1^)^a^	IC_50_ (μM)^a^
	500	250	125	62.50	31.25	15.60	7.80	3.90	2	1	0		
**5a**	5.19	11.84	25.06	37.15	48.91	62.87	80.96	91.43	97.02	100	100	29.90	80.06
**5b**	5.38	11.97	24.02	36.59	47.38	63.20	78.15	87.56	94.03	98.76	100	28.60	70.88
**5c**	4.31	8.65	16.37	28.74	40.96	58.71	73.04	89.21	96.28	100	100	23.20	59.87
**5d**	2.65	5.34	10.26	19.45	28.73	39.04	57.26	71.89	85.22	92.34	100	10.90	26.72
**5e**	2.94	6.71	14.09	23.87	36.29	49.85	70.63	89.24	96.31	99.72	100	15.50	34.26
**5f**	4.23	9.86	20.15	32.76	45.13	59.38	75.04	82.38	91.75	97.43	100	25.80	57.91
**5g**	2.75	6.39	10.42	18.65	28.91	40.74	56.26	70.89	83.11	88.62	100	10.90	26.11
**5h**	2.86	5.43	12.87	22.96	34.53	47.28	65.39	80.72	89.86	97.13	100	14.40	34.41
**5i**	4.73	10.59	18.42	28.96	40.67	59.23	73.18	85.21	92.74	97.35	100	23.30	62.06
**5j**	2.98	6.74	10.85	19.73	26.8	37.28	49.06	70.31	84.15	91.38	100	7.60	18.74
**5k**	8.92	16.34	24.08	35.17	49.01	72.38	89.42	98.36	100	100	100	30.50	78.31
**5l**	1.89	4.37	8.76	19.04	30.63	43.86	59.12	76.44	90.63	96.42	100	12.40	30.25
**5m**	6.72	10.86	22.38	31.49	46.75	69.42	86.03	92.34	99.71	100	100	28.90	63.61
**5n**	4.86	9.73	19.48	31.72	45.29	64.18	80.63	92.34	97.23	100	100	27.30	61.00
**5o**	2.89	6.54	15.93	24.06	36.41	52.97	68.02	79.19	91.42	98.76	100	18.30	43.63
**5p**	3.69	8.71	18.63	29.46	41.87	54.06	71.32	87.14	95.20	99.73	100	20.70	49.23
**6a**	3.65	8.29	16.34	25.87	37.06	46.92	58.43	80.71	87.53	93.14	100	13.50	36.15
**6b**	6.14	13.65	25.38	37.25	56.41	79.84	92.36	98.6	100	100	100	41.70	103.35
**6c**	3.74	7.46	15.28	27.4	41.35	56.79	68.42	82.37	91.43	97.60	100	22.40	57.81
**6d**	4.08	6.82	11.43	19.46	30.67	43.20	59.13	71.44	85.12	92.37	100	12.20	29.91
**6e**	4.62	11.29	20.47	34.13	45.29	62.37	80.94	89.76	97.02	100	100	26.80	59.24
**6f**	6.31	13.45	19.75	31.42	47.23	71.94	89.56	97.13	100	100	100	29.40	65.99
**6g**	9.56	21.87	30.64	43.19	59.46	78.28	91.40	98.76	100	100	100	49.40	118.33
**6h**	1.98	4.87	9.72	19.93	28.61	40.72	63.18	79.43	90.64	97.39	100	12.30	29.39
**6i**	7.94	11.52	24.43	36.25	49.72	71.49	89.70	97.41	100	100	100	30.90	82.30
**6j**	3.87	7.96	16.20	27.85	41.79	54.03	71.48	88.60	96.23	100	100	20.70	51.05
**6k**	7.18	12.98	23.06	33.97	47.85	63.28	78.91	90.68	97.89	100	100	29.00	74.46
**6l**	6.74	14.35	21.88	35.46	46.29	58.17	78.03	86.36	94.12	98.78	100	26.30	64.16
**6m**	4.28	9.53	18.75	31.92	40.06	47.41	73.65	90.37	98.91	100	100	14.80	32.57
**6n**	11.82	23.48	30.51	41.3	47.42	60.31	82.42	97.14	100	100	100	28.07	62.72
**6o**	31.96	49.72	73.68	90.63	97.46	100	100	100	100	100	100	248.00	591.25
**6p**	2.79	6.28	13.91	25.04	37.18	52.94	68.29	84.67	92.84	98.25	100	18.50	44.00
5-FU	10.28	19.45	25.39	39.48	57.21	70.82	86.19	94.36	99.25	100	100	43.9	337.48

IC_50_ value: concentration causing 50% inhibition of cell viability

^a^Mean of three results obtained from three experiments

**Table 3:** In vitro anticancer screening of the synthesized compounds against human breast carcinoma cell line (MCF-7)

Comp. no.	Validity for sample conc.											IC_50_ (μg mL^−1^)^a^	IC_50_ (μM)^a^
	500	250	125	62.50	31.25	15.60	7.80	3.90	2	1	0		
**5a**	7.34	14.03	29.47	41.58	54.29	71.43	87.50	96.75	100	100	100	41.80	111.92
**5b**	7.13	13.42	30.96	42.37	56.29	68.41	83.77	91.40	98.32	100	100	45.40	112.52
**5c**	5.28	11.32	20.79	32.65	48.51	70.38	86.42	94.03	99.71	100	100	30.10	77.68
**5d**	3.46	7.28	15.09	27.41	36.27	45.62	63.18	80.94	93.46	99.53	100	13.70	33.59
**5e**	4.02	9.56	18.48	29.67	40.82	54.61	73.28	91.42	98.70	100	100	20.80	45.98
**5f**	6.17	14.38	27.56	39.4	53.29	68.43	81.70	93.68	99.85	100	100	38.70	86.86
**5g**	3.86	8.24	15.37	24.16	32.95	41.68	49.80	67.48	85.26	93.84	100	7.76	18.59
**5h**	3.94	7.81	16.23	31.42	39.79	48.20	67.41	82.76	91.30	98.72	100	14.90	35.61
**5i**	6.28	13.47	25.13	36.78	48.50	67.41	81.67	89.43	98.16	100	100	30.00	79.91
**5j**	4.17	9.82	18.78	27.05	35.23	46.19	54.82	69.46	87.34	94.29	100	12.10	29.84
**5k**	13.49	21.86	32.75	46.23	57.18	76.94	88.60	97.41	100	100	100	51.70	132.74
**5l**	2.73	6.46	11.38	24.95	34.89	42.67	51.53	65.76	83.20	91.42	100	9.140	22.30
**5m**	9.85	18.2	30.67	42.96	51.78	68.92	84.68	95.41	99.62	100	100	37.50	82.54
**5n**	7.63	15.26	27.39	38.04	46.15	58.20	71.36	88.42	96.28	100	100	26.20	58.55
**5o**	3.46	9.82	20.31	29.57	43.60	56.89	70.42	83.97	92.40	97.36	100	23.60	56.26
**5p**	4.98	11.75	21.42	36.75	50.38	69.41	85.26	93.02	99.76	100	100	32.10	76.35
**6a**	4.94	10.73	23.4	32.79	45.17	56.24	71.38	85.02	92.47	98.25	100	24.40	65.33
**6b**	8.71	20.42	37.53	49.81	62.97	85.46	93.04	99.32	100	100	100	62.00	153.66
**6c**	5.31	9.14	19.56	32.71	49.82	70.38	81.6	92.88	98.76	100	100	31.10	80.26
**6d**	6.79	13.4	19.85	27.93	36.7	48.61	63.87	76.45	88.29	96.36	100	14.90	36.53
**6e**	7.46	19.53	28.65	40.37	52.91	69.42	82.36	91.73	98.6	100	100	38.50	85.11
**6f**	9.56	17.28	28.67	36.7	50.98	67.39	82.15	93.69	98.72	100	100	33.40	74.97
**6g**	15.68	27.83	38.17	52.46	69.9	83.51	92.78	99.52	100	100	100	73.30	175.58
**6h**	2.37	7.54	15.18	23.65	34.89	45.13	60.97	75.86	87.41	95.64	100	13.20	31.54
**6i**	11.76	21.49	34.85	43.96	58.28	77.39	91.47	97.92	100	100	100	49.30	131.31
**6j**	5.92	11.48	23.69	34.73	47.21	68.46	80.93	92.64	98.23	100	100	29.14	71.87
**6k**	12.41	20.53	30.78	39.62	56.34	71.48	86.24	98.4	100	100	100	43.00	110.41
**6l**	10.32	19.67	32.94	41.78	51.85	65.04	79.12	90.65	97.54	100	100	36.90	90.02
**6m**	7.64	16.29	24.31	35.17	45.04	56.28	69.46	82.78	94.13	99.2	100	24.30	53.48
**6n**	19.47	31.85	40.91	49.76	58.49	71.32	86.95	97.81	100	100	100	61.60	137.65
**6o**	37.04	52.31	76.45	91.32	98.74	100	100	100	100	100	100	287.00	684.23
**6p**	3.45	9.74	20.37	31.96	46.29	70.34	85.21	93.18	99.4	100	100	28.70	68.26
5-FU	9.18	17.84	28.01	35.39	47.13	60.35	71.82	86.97	95.23	98.12	100	27.80	213.71

IC_50_ value: concentration causing 50% inhibition of cell viability

^a^Mean of three results obtained from three experiments

**Fig. 3:**
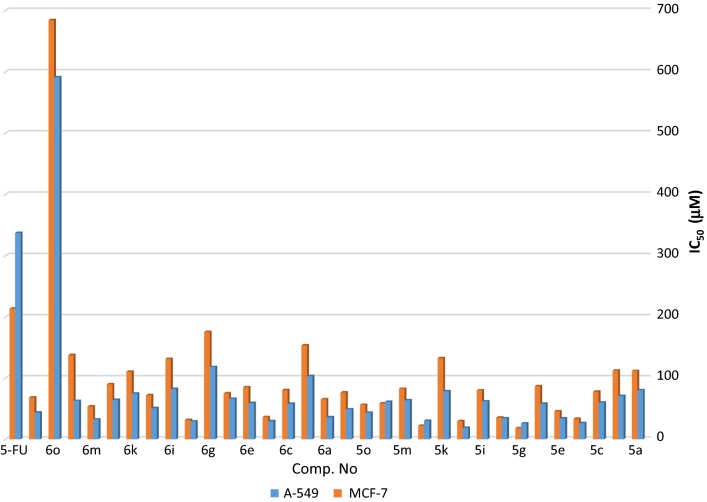
Comparison of cytotoxic activity of the tested compounds against (A-549) and (MCF-7) cell lines

From the obtained results in Tables 2 and 3, we can show the effect of the following compounds on A-549 and MCF-7 cancer cell lines, respectively. Compound **5j** possessing 4-methoxyphenylamino acetamide of morpholinosulfonyl moiety exhibited IC_50_ value of 18.74 µM and 29.84 µM, respectively; Compound **5g** having 4-carboxyphenylamino acetamide of piperidinosulfonyl moiety showed IC_50_ value of 26.11 µM and 18.59 µM, respectively. While, compound **5d** having 4-chlorophenylamino acetamide of piperidinosulfonyl moiety showed IC_50_ value of 26.72 µM and 33.59 µM, respectively. On the other hand, compound **6h** having 4-nitrophenyl acetamide of piperidinosulfonyl moiety showed IC_50_ value of 29.39 µM and 31.54 µM, respectively. Furthermore, compound **6d** having 4-chlorophenyl acetamide of piperidinosulfonyl moiety showed IC_50_ value of 29.91 µM and 36.53 µM, respectively. Finally, compound **5l** having 4-chlorophenylamino acetamide of morpholinosulfonyl moiety showed IC_50_ value of 30.25 µM and 22.30 µM, respectively. From the data represented in Table 2 and Fig. 3 it is clear that, the cytotoxic activity order against cell line (A-549) having the following order: **5j** > **5g** > **5d** > **6h** > **6d** > **5l** > **6m** > **5e** > **5h** > **6a** > **5o** > **6p** > **5p** > **6j** > **6c** > **5f** > **6e** > **5c** > **5n** > **5i** > **6n** > **5m** > **6l** > **6f** > **5b** > **6k** > **5k** > **5a** > **6i** > **6b** > **6g** > **5-FU** > **6o**. However, from data shown in Table 3 and Fig. 3, we can concluded that, the cytotoxic activity order against cell line (MCF-7) is: **5g** >** 5l** > **5j** > **6h** > **5d** > **5h** > **6d** > **5e** > **6m** > **5o** > **5n** > **6a** > **6p** > **6j** > **6f** > **5p** > **5c** > **5i** > **6c** > **5m** > **6e** > **5f** > **6l** > **6k** > **5a** > **5b** > **6i** > **5k** > **6n** > **6b** > **6g** > **5-FU** > **6o**. The previous biological screening results of the tested compounds lead to development of potential anticancer agents.

### Docking and molecular modeling study

**Fig. 4:**
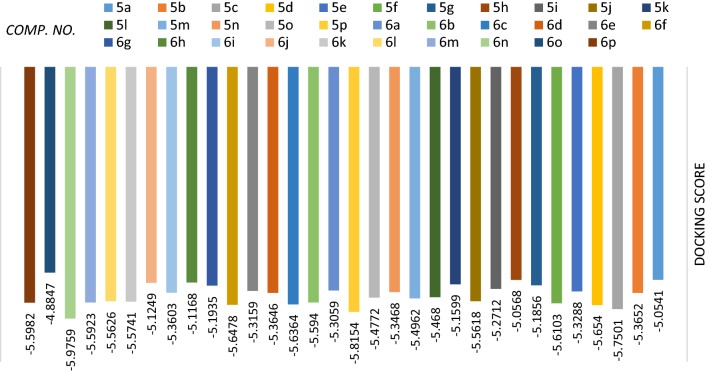
The docking score energy of the tested synthesized compounds

The best enzymes which involved in the improvement of anticancer and antimicrobial activity are thymidylate synthase and dihydrofolate reductase (DHFR) [50, 54]. In the present investigation, Molecular Operating Environment (MOE) [55] module was accomplished to vindicate the cytotoxic potency of all tested compounds. Furthermore, study of Molecular docking help in explanation of how compounds act through their reaction with the enzyme active sites. Docking was performed for the compounds **5a**–**5p** and **6a**–**6p** on the (DHFR) to predict their action as anticancer drugs (c.f. Additional file 1). The synthesized compounds show numerous interactions with DHFR enzyme. It’s important to mention that compounds **6n**, **5p**, **5c**, **5d**, **6f** and **6c** could make their action via inhibition of the DHFR enzyme (Table 4). In Fig. 4, the docking score energy for the newly synthesized compounds was indicated as the following order: **6n **>** 5p **>** 5c **>** 5d **>** 6f **>** 6c **>** 5f **>** 6p **>** 6b **>** 6m **>** 6k **>** 6l **>** 5j **>** 5m **>** 5o **>** 5l **>** 5b **>** 6d **>** 6i **>** 5n **>** 5e **>** 6e **>** 6a **>** 5i **>** 6g **>** 5g **>** 5k **>** 6j **>** 6h **>** 5h **>** 5a **>** 6o**.

**Table 4:** Score energy of the tested compounds **5a**–**p** and **6a**–**p**

Comp. no.	Score	E_conf	E_place	E_score1	E_score2	E_refine
**5a**	− 5.0541	26.6140	− 31.9343	− 6.5932	− 5.0541	− 24.1143
**5b**	− 5.3652	15.3242	− 38.0154	− 6.0735	− 5.3652	− 26.8906
**5c**	− 5.7501	17.6782	− 22.8678	− 6.5044	− 5.7501	− 30.5432
**5d**	− 5.6540	24.5859	− 31.2672	− 6.5266	− 5.6540	− 27.5442
**5e**	− 5.3288	23.5121	− 39.4718	− 6.5074	− 5.3288	− 27.0244
**5f**	− 5.6103	33.4059	− 20.9497	− 6.4211	− 5.6103	− 29.1261
**5g**	− 5.1856	− 42.3167	− 22.2309	− 6.4648	− 5.1856	− 24.4937
**5h**	− 5.0568	49.2723	− 4.9075	− 5.7500	− 5.0568	− 23.4996
**5i**	− 5.2712	59.3063	− 22.4680	− 5.4465	− 5.2712	− 24.9909
**5j**	− 5.5618	48.5778	− 27.9431	− 6.3215	− 5.5618	− 28.8293
**5k**	− 5.1599	55.1210	− 25.0222	− 6.7661	− 5.1599	− 25.1400
**5l**	− 5.4680	52.7716	− 29.2492	− 6.7614	− 5.4680	− 27.0499
**5m**	− 5.4962	54.0440	− 28.8482	− 5.9473	− 5.4962	− 27.2928
**5n**	− 5.3468	71.7895	− 10.6748	− 5.9170	− 5.3468	− 25.8020
**5o**	− 5.4772	− 8.5979	− 36.7758	− 6.7380	− 5.4772	− 29.3019
**5p**	− 5.8154	93.6886	− 29.1507	− 6.7008	− 5.8154	− 27.9044
**6a**	− 5.3059	28.9177	− 33.5175	− 6.5957	− 5.3059	− 25.2110
**6b**	− 5.5940	26.3542	− 40.3486	− 7.4533	− 5.5940	− 27.7459
**6c**	− 5.6364	21.9008	− 5.0259	− 6.0110	− 5.6364	− 26.9364
**6d**	− 5.3646	22.8002	− 5.5670	− 6.3649	− 5.3646	− 27.1061
**6e**	− 5.3159	23.2172	− 28.6743	− 6.2439	− 5.3159	− 26.9349
**6f**	− 5.6478	35.7362	− 29.6484	− 6.7947	− 5.6478	− 28.8622
**6g**	− 5.1935	− 37.9832	− 17.5087	− 4.9311	− 5.1935	− 26.2035
**6h**	− 5.1168	54.5965	− 35.3328	− 7.2622	− 5.1168	− 24.2974
**6i**	− 5.3603	64.5399	− 47.4900	− 6.8921	− 5.3603	− 26.9684
**6j**	− 5.1249	55.1297	− 29.5479	− 6.4586	− 5.1249	− 25.9026
**6k**	− 5.5741	58.0647	− 33.7004	− 6.4011	− 5.5741	− 29.5841
**6l**	− 5.5626	61.7654	− 25.5315	− 6.9615	− 5.5626	− 30.0500
**6m**	− 5.5923	61.2420	− 21.5826	− 6.5719	− 5.5923	− 29.0718
**6n**	− 5.9759	67.6646	− 8.4886	− 6.9574	− 5.9759	− 32.6043
**6o**	− 4.8847	− 4.0898	− 9.1947	− 5.7962	− 4.8847	− 21.8406
**6p**	− 5.5982	96.4896	− 35.1380	− 7.6471	− 5.5982	− 28.5998

*Score* lower scores shows more poses that are favorable. The unit is kcal mol^−1^; *E-conf* indicate the energy of the conformer; *E-place* score from the placement stage; *E-score 1* score from the first rescoring stage; *E-score 2* score from the second rescoring stage; *E-refine* score from the refinement stage

#### Docking of 5-Fluorouracil (5-FU) into DHFR

**Fig. 5:**
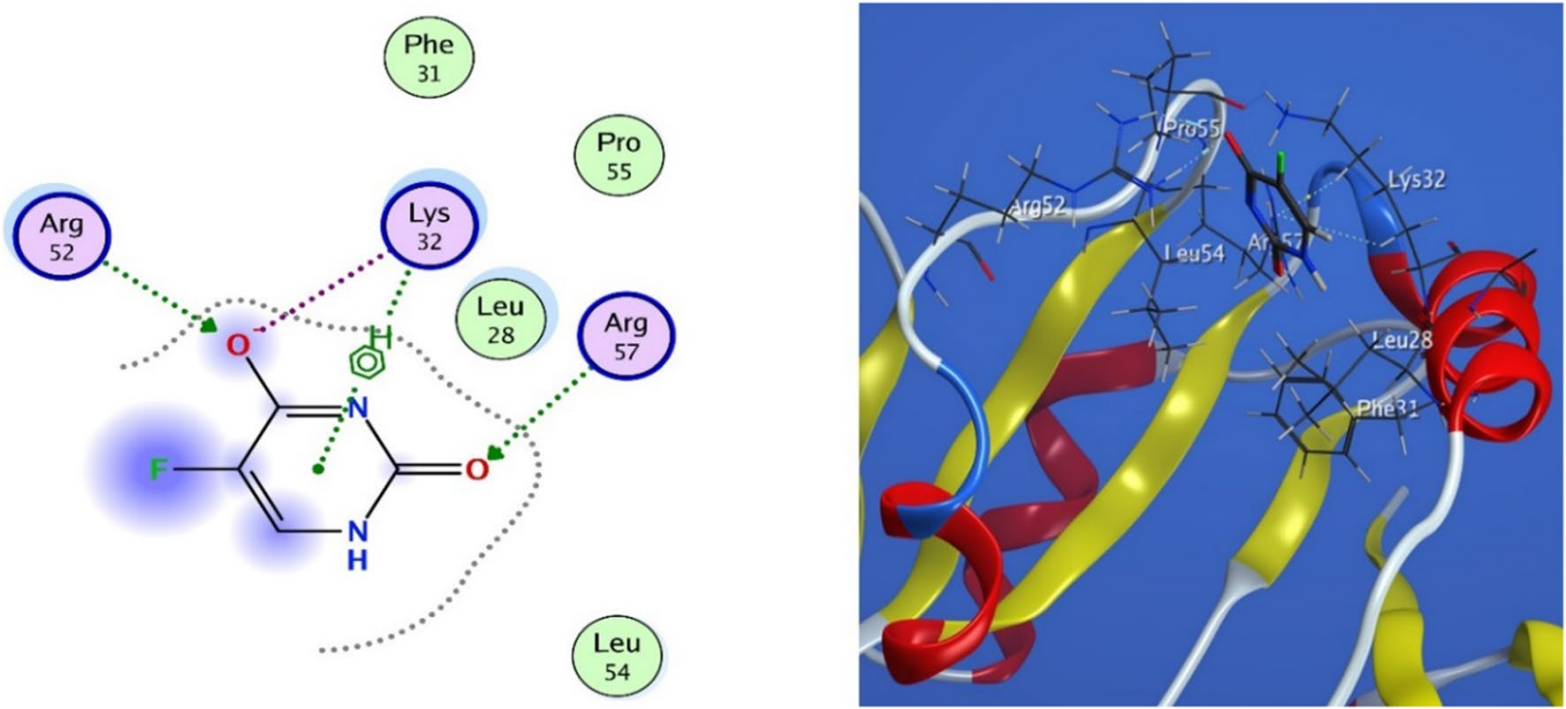
Docking of (5-FU) into DHFR

The docking studies at the active site showed presence of two hydrogen bond interactions as two oxygen atoms acted as hydrogen bond acceptors with Arg 52 and Arg 57 (3.13 Å and 2.96 Å) with binding energies of − 2.8 and − 6.5 kcal mol^−1^, respectively. Moreover, it indicated the presence of two ionic bond interactions that is between oxygen atom and amino acid residues Lys 32 and Arg 52 (3.14 Å and 3.05 Å) with binding energies of − 3.6 and − 4.1 kcal mol^−1^, respectively. Furthermore, it showed an arene-H interaction between the phenyl ring and Lys 32 (3.81 Å) with binding energy of − 0.9 kcal mol^−1^. besides, many hydrophobic interactions with: Leu 28, Leu 54, Phe 31 and Pro 55, (Fig. 5).

#### Docking of compounds **5g**, **5j**, **6d** and **6h** into DHFR

**Fig. 6:**
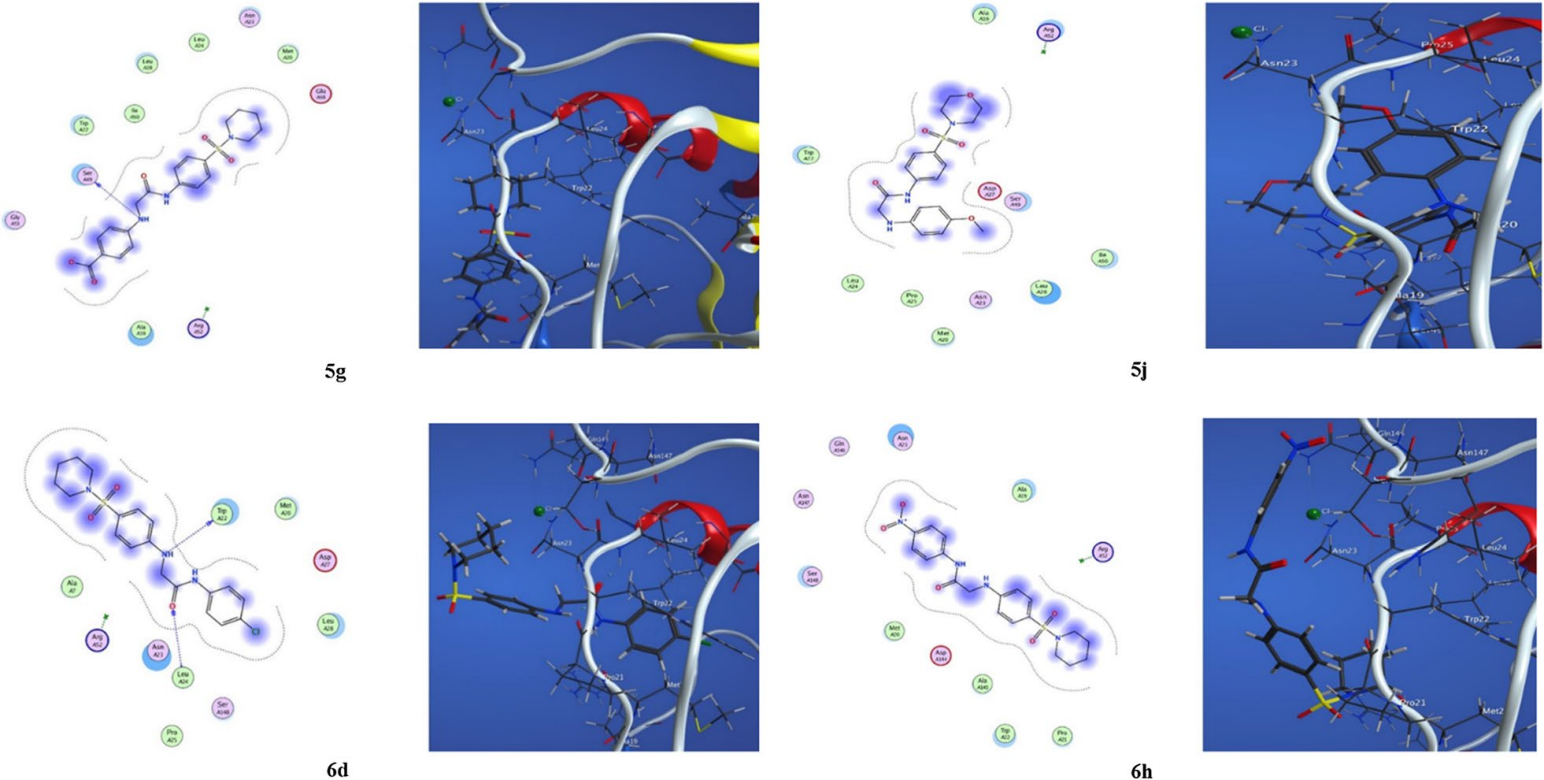
Docking of compounds **5g**, **5j**, **6d** and **6h** into DHFR

Methotrexate (PDB ID: 4DFR) used as a template with dihydrofolate reductase co-crystallized in MOE docking studies for the inhibitors. Docking of **5g** showed one hydrogen bond interaction as one of the nitrogen atom acted as a hydrogen bond donor with Ser 49 (3.04 Å) with energy − 0.9 kcal mol^−1^. This is beside several hydrophobic interactions with various amino acid residues: Glu 48, Met 20, Asn 23, Leu 24, Leu 28, Ile 50, Trp 22, Gly 51, Ala 19, Arg 52. In addition, molecular docking study of **5j** indicated hydrophobic interactions between various atoms and amino acid residues: Asp 27, Ser 49, Ile 50, Leu 28, Asn 23, Met 20, Pro 25, Leu 24, Trp 22, Ala 19, Arg 52. Furthermore, docking studies of **6d** showed that nitrogen acted as a hydrogen bond donor with Trp 22 (2.97 Å) with binding energy of − 1.7 kcal mol^−1^. Whereas oxygen of carbonyl group acted as a hydrogen bond acceptor with Leu 24 (3.07 Å) with binding energy of − 0.7 kcal mol^−1^. This is beside hydrophobic interactions different amino acid residues: Met 20, Asp 27, Leu 28, Ser 148, Pro 25, Asn 23, Arg 52, Ala 7. While, docking of **6h** showed that there are various hydrophobic interactions among atoms of the compound and different amino acid residues: Arg 52, Pro 21, Trp 22, Ala 145, Asp 144, Met 20, Ser 148, Asn 147, Gln 146, Asn 23, Ala 19 (Fig. 6).

## Conclusion

We report herein the synthesis of some new series of 2-(arylamino)acetamides and *N*-arylacetamides bearing sulfonamide moieties. Most of these new compounds exhibited significant anticancer activity against human lung carcinoma (A-549) and human breast carcinoma (MCF-7) cell lines, when compared to 5-Fluorouracil as a reference drug. In addition, on antimicrobial evaluation; some of these synthesized compounds showed remarkable activity as antibacterial and antifungal agents. To the best of our knowledge, these multi-addressable properties of the new synthesized sulfonamides reported in this work will open a new era in the field of medicinal chemistry and can be considered as pharmacophores.

## Experimental

### Chemistry

#### General methods

All solvents used purchased from Sigma-Aldrich are spectroscopic grade and used without further purifications. Melting points were determined on a Stuart SMP3 melting point apparatus and are uncorrected. FT-IR spectra were recorded on a Shimadzu IR-3600 FT-IR spectrometer in KBr pellets. NMR spectra were acquired on a Bruker Avance 500 instrument (500 MHz for ^1^H, 125 MHz for ^13^C) in CDCl_3_ and DMSO-*d*_*6*_ solutions, using residual solvent signals as internal standards.

### General procedure for synthesis of 2-chloro-*N*-(4-((piperidino/morpholino)sulfonyl)-phenyl)acetamides **2a,b**

A mixture of 4-(piperidin-1-ylsulfonyl)aniline (**1a**) or 4-(morpholin-4-ylsulfonyl)aniline (**1b**) **1** (0.1 mol) and chloroacetyl chloride (8.0 mL, 0.1 mol) in DMF (20 mL) was stirred at room temperature for 2 h. The reaction mixture was poured onto ice-water. The solid obtained was filtered off and crystallized from ethanol to give **2a,b**.

#### 2-Chloro-*N*-(4-(piperidin-1-ylsulfonyl)phenyl)acetamide (**2a**)

White crystals, yield (98%), m.p. 162–163 °C. FT-IR: 3334 (NH), 3056 (CH arom.), 2945 (CH aliph.), 1696 (C=O) cm^−1^. ^1^H NMR (CDCl_3_): *δ* = 8.56 (s, 1H, NH), 7.76 (d, *J *= 5.5 Hz, 2H, Ph-H), 7.73 (d, *J* = 5.5 Hz, 2H, Ph-H), 4.21 (s, 2H, CH_2_), 3.00–2.97 (m, 4H, 2CH_2_), 1.66–1.64 (m, 4H, 2CH_2_), 1.43–1.42 (m, 2H, CH_2_) ppm. ^13^C NMR: *δ* = 164.4 (C=O), 140.7 (C), 132.0 (C), 128.9 (2CH), 119.7 (2CH), 46.9 (CH_2_), 42.9 (2CH_2_), 25.1 (2CH_2_), 23.4 (CH_2_) ppm.

#### 2-Chloro-*N*-(4-(morpholinosulfonyl)phenyl)acetamide (**2b**)

White crystals, yield (96%), m.p. 189–190 °C. FT-IR: 3336 (NH), 3050 (CH arom.), 2961 (CH aliph.), 1694 (C=O) cm^−1^. ^1^H NMR (CDCl_3_): *δ* = 8.53 (s, 1H, NH), 7.79 (d, *J* = 5.0 Hz, 2H, Ph-H), 7.75 (d, *J* = 5.0 Hz, 2H, Ph-H), 4.24 (s, 2H, CH_2_), 3.75–3.73 (m, 4H, 2CH_2_), 3.00–2.90 (m, 4H, 2CH_2_) ppm. ^13^C NMR: *δ*=164.3 (C=O), 141.1 (C), 130.9 (C), 130.0 (CH), 129.1 (CH), 119.8 (CH), 114.0 (CH), 66.1 (CH_2_), 46.0 (2CH_2_), 42.8 (2CH_2_) ppm.

### General procedure for the synthesis of 2-chloro-*N*-arylacetamides **4a**–**h**

A mixture of different aryl amines namely; aniline, 4-methoxyaniline, 4-methylaniline, 4-chloroaniline, 4-bromoaniline, ethyl 4-aminobenzoate, 4-aminobenzoic acid, and 4-nitroaniline (0.05 mol) and chloroacetyl chloride (4.0 ml, 0.05 mol) in DMF (10 mL) was stirred at room temperature for 2 h. The reaction mixture was poured onto ice–water. The solid obtained was filtered off, dried and crystallized from dioxane to give **4a**–**i**. The physical and chemical properties of **4a**–**i** were matched as previously reported [56–61].

### General procedure for synthesis of 2-(arylamino)-*N*-(4-(piperidin-1-ylsulfonyl)-phenyl)acetamides **5a**–**h** and 2-(arylamino)-*N*-(4-(morpholino-sulfonyl)phenyl)-acetamides **5i**–**p**

A mixture of chloro compound **2a**,**b** (0.001 mol) and different aryl amines namely; aniline, 4-methoxyaniline, 4-methylaniline, 4-chloroaniline, 4-bromoaniline, ethyl 4-aminobenzoate, 4-aminobenzoic acid, and 4-nitroaniline (0.001 mol) in absolute ethanol (20 mL) was refluxed for 3–5 h. The reaction mixture was concentrated under reduced pressure, the solid obtained was filtered, washed with *n*-hexane, dried and recrystallized from ethanol to give the titled products **5a**–**h** and **5i**–**p**.

#### 2-(Phenylamino)-*N*-(4-(piperidin-1-ylsulfonyl)phenyl)acetamide (**5a**)

Beige crystals, yield (60%), m.p. 115–116 °C. FT-IR: 3378 (NH), 3311 (NH), 3090 (CH arom.), 2940 (CH aliph.), 2850 (CH aliph.), 1679 (C=O) cm^−1^. ^1^H-NMR (DMSO-*d*_*6*_): *δ* = 10.81 (s, 1H, NH), 10.51 (s, 1H, NH), 7.88 (d, *J* = 11.0 Hz, 2H, Ph-H), 7.69 (d, *J* = 11.0 Hz, 2H, Ph-H), 7.13-7.09 (m, 2H, Ph-H), 6.65–6.61 (m, 3H, Ph-H), 4.33 (s, 2H, CH_2_), 2.85–2.83 (m, 4H, 2CH_2_), 1.54–1.52 (m, 4H, 2CH_2_), 1.32–1.30 (m, 2H, CH_2_) ppm. ^13^C-NMR (DMSO-*d*_*6*_): *δ* = 165.8 (C=O), 143.3 (C), 142.9 (C), 130.2 (C), 129.4 (CH), 129.2 (CH), 129.1 (CH), 119.6 (CH), 119.4 (CH), 113.1 (CH), 47.0 (CH_2_), 44.0 (CH_2_), 24.6 (CH_2_), 22.8 (CH_2_) ppm.

#### 2-(4-Methoxyphenylamino)-*N*-(4-(piperidin-1-ylsulfonyl)phenyl)acetamide (**5b**)

Pale yellow crystals, yield (69%), m.p. 85–87 °C. FT-IR: 3442 (NH), 3299 (NH), 3043 (CH arom.), 2940 (CH aliph.), 2850 (CH aliph.), 1681 (C=O) cm^−1^. ^1^H-NMR (DMSO-*d*_*6*_): *δ* = 10.75 (s, 1H, NH), 10.52 (s, 1H, NH), 7.94 (d, *J* = 10.5 Hz, 2H, Ph-H), 7.72 (d, *J* = 10.5 Hz, 2H, Ph-H), 6.77 (d, *J* = 10.5 Hz, 2H, Ph-H), 6.68 (d, 2H, *J* = 10.5 Hz, Ph-H), 4.33 (s, 2H, CH_2_), 3.93 (s, 3H, CH_3_), 2.85–2.84 (m, 4H, 2CH_2_), 1.53–1.51 (m, 4H, 2CH_2_), 1.32–1.30 (m, 2H, CH_2_) ppm. ^13^C-NMR (DMSO-*d*_*6*_): *δ* = 165.8 (C=O), 143.1 (C), 142.9 (C), 132.9 (C), 132.5 (C), 129.2 (CH), 124.2 (CH), 119.6 (CH), 115.1 (CH), 55.7 (CH_3_), 47.0 (CH_2_), 43.8 (CH_2_), 25.1 (CH_2_), 23.3 (CH_2_) ppm.

#### *N*-(4-(Piperidin-1-ylsulfonyl)phenyl)-2-(*p*-tolylamino)acetamide (**5c**)

Beige crystals, yield (67%), m.p. 100–102 °C. FT-IR: 3378 (NH), 3310 (NH), 3080 (CH arom.), 2942 (CH aliph.), 2850 (CH aliph.), 1679 (C=O) cm^−1^. ^1^H-NMR (DMSO-*d*_*6*_): *δ* = 10.75 (s, 1H, NH), 10.40 (s, 1H, NH), 7.86 (d, *J* = 11.0 Hz, 2H, Ph-H), 7.83 (d, *J* = 11.0 Hz, 2H, Ph-H), 7.70 (d, *J* = 10.5 Hz, 2H, Ph-H), 6.66 (d, 2H, *J* = 10.5 Hz, Ph-H), 4.32 (s, 2H, CH_2_), 2.86–2.83 (m, 4H, 2CH_2_), 2.14 (s, 3H, CH_3_), 1.52–1.51 (m, 4H, 2CH_2_), 1.34–1.31 (m, 2H, CH_2_) ppm. ^13^C-NMR (DMSO-*d*_*6*_): *δ* = 165.6 (C=O), 146.3 (C), 142.9 (C), 130.3 (C), 130.0 (C), 129.8 (CH), 129.2 (CH), 119.5 (CH), 113.0 (CH), 47.0 (CH_2_), 44.0 (CH_2_), 25.1 (CH_2_), 23.3 (CH_2_), 20.5 (CH_3_) ppm.

#### 2-(4-Chlorophenylamino)-*N*-(4-(piperidin-1-ylsulfonyl)phenyl)acetamide (**5d**)

White crystals, yield (58%), m.p. 77–79 °C. FT-IR: 3334 (NH), 3329 (NH), 3056 (CH arom.), 2943 (CH aliph.), 2853 (CH aliph.), 1698 (C=O) cm^−1^. ^1^H-NMR (DMSO-*d*_*6*_): *δ* = 10.82 (s, 1H, NH), 10.52 (s, 1H, NH), 7.85 (d, *J* = 11.0 Hz, 2H, Ph-H), 7.70 (d, *J* = 11.0 Hz, 2H, Ph-H), 7.12 (d, *J* = 10.0 Hz, 2H, Ph-H), 6.61 (d, *J* = 10.0 Hz, 2H, Ph-H), 4.33 (s, 2H, CH_2_), 2.86–2.84 (m, 4H, 2CH_2_), 1.53–1.51 (m, 4H, 2CH_2_), 1.35–1.33 (m, 2H, CH_2_) ppm. ^13^C-NMR (DMSO-*d*_*6*_): *δ *= 165.8 (C=O), 147.7 (C), 143.0 (C), 142.9 (C), 130.2 (C), 129.2 (CH), 119.6 (CH), 119.4 (CH), 114.1 (CH), 47.0 (CH_2_), 44.0 (CH_2_), 25.1 (CH_2_), 23.3 (CH_2_) ppm.

#### 2-(4-Bromophenylamino)-*N*-(4-(piperidin-1-ylsulfonyl)phenyl)acetamide (**5e**)

Pale yellow crystals, yield (50%), m.p. 84–86 °C. FT-IR: 3499 (NH), 3327 (NH), 3056 (CH arom.), 2943 (CH aliph.), 2854 (CH aliph.), 1696 (C=O) cm^−1^. ^1^H-NMR (DMSO-*d*_*6*_): *δ* = 10.88 (s, 1H, NH), 10.56 (s, 1H, NH), 7.85 (d, *J* = 10.5 Hz, 2H, Ph-H), 7.70 (d, *J* = 10.5 Hz, 2H, Ph-H), 7.20 (d, *J* = 10.0 Hz, 2H, Ph-H), 6.58 (d, *J* = 10.0 Hz, 2H, Ph-H), 4.33 (s, 2H, CH_2_), 2.86–2.84 (m, 4H, 2CH_2_), 1.54–1.52 (m, 4H, 2CH_2_), 1.36–1.34 (m, 2H, CH_2_) ppm. ^13^C-NMR (DMSO-*d*_*6*_): *δ* = 165.4 (C=O), 147.2 (C), 142.5 (C), 132.2 (C), 131.6 (C), 129.8 (CH), 128.7 (CH), 119.1 (CH), 114.8 (CH), 46.6 (CH_2_), 43.5 (CH_2_), 24.6 (CH_2_), 22.8 (CH_2_) ppm.

#### Ethyl 4-(2-oxo-2-(4-(piperidin-1-ylsulfonyl)phenylamino)ethylamino)benzoate (**5f**)

Beige crystals, yield (75%), m.p. 150–151 °C. FT-IR: 3485 (NH), 3349 (NH), 3054 (CH arom.), 2944 (CH aliph.), 2855 (CH aliph.), 1699 (C=O), 1690 (C=O) cm^−1^. ^1^H-NMR (DMSO-*d*_*6*_): *δ* = 10.93 (s, 1H, NH), 10.69 (s, 1H, NH), 7.88 (d, *J* = 10.5 Hz, 2H, Ph-H), 7.75–7.66 (m, 6H, Ph-H), 4.34 (s, 2H, CH_2_), 4.21 (q, *J* = 8.5 Hz, 2H, CH_2_), 2.85-2.83 (m, 4H, 2CH_2_), 1.52–1.50 (m, 4H, 2CH_2_), 1.33–1.29 (m, 2H, CH_2_), 1.26 (t, *J* = 8.5 Hz, 3H, CH_3_) ppm. ^13^C-NMR (DMSO-*d*_*6*_): *δ* = 169.8 (C=O), 165.8 (C=O), 152.8 (C), 143.1 (C), 143.0 (C), 131.4 (CH), 130.2 (C), 129.2 (CH), 119.6 (CH), 115.7 (CH), 60.3 (CH_2_), 47.0 (CH_2_), 44.0 (CH_2_), 25.1 (CH_2_), 23.3 (CH_2_), 14.7 (CH_3_) ppm.

#### 4-(2-Oxo-2-(4-(piperidin-1-ylsulfonyl)phenylamino)ethylamino)benzoic acid (**5g**)

Beige crystals, yield (57%), m.p. 174–175 °C. FT-IR: 3380 (br, OH), 3333 (NH), 3062 (CH arom.), 2941 (CH aliph.), 2852 (CH aliph.), 1700 (C=O), 1680 (C=O) cm^−1^. ^1^H-NMR (DMSO-*d*_*6*_): *δ* = 10.81 (s, 1H, NH), 10.66 (s, 1H, NH), 7.84 (d, *J* = 10.5 Hz, 2H, Ph-H), 7.72–7.68 (m, 4H, Ph-H), 6.63 (d, *J* = 10.5 Hz, 2H, Ph-H), 4.85 (s, 1H, OH), 4.33 (s, 2H, CH_2_), 2.86–2.84 (m, 4H, 2CH_2_), 1.54–1.52 (m, 4H, 2CH_2_), 1.35–1.33 (m, 2H, CH_2_) ppm. ^13^C-NMR (DMSO-*d*_*6*_): *δ* = 166.2 (C=O), 165.8 (C=O), 142.9 (C), 142.8 (C), 132.1 (C), 131.9 (CH), 130.2 (C), 129.2 (CH), 119.8 (CH), 113.5 (CH), 47.0 (CH_2_), 44.0 (CH_2_), 25.1 (CH_2_), 23.3 (CH_2_) ppm.

#### 2-(4-Nitrophenylamino)-*N*-(4-(piperidin-1-ylsulfonyl)phenyl)acetamide (**5h**)

Yellow crystals, yield (71%), m.p. 108–110 °C. FT-IR: 3483 (NH), 3363 (NH), 3057 (CH arom.), 2945 (CH aliph.), 2857 (CH aliph.), 1699 (C=O) cm^−1^. ^1^H-NMR (DMSO-*d*_*6*_): *δ* = 10.75 (s, 1H, NH), 10.10 (s, 1H, NH), 7.94 (d, *J* = 11.0 Hz, 2H, Ph-H), 7.84 (d, *J* = 10.5 Hz, 2H, Ph-H), 7.70 (d, *J* = 10.5 Hz, 2H, Ph-H), 6.60 (d, *J* = 11.0 Hz, 2H, Ph-H), 4.32 (s, 2H, CH_2_), 2.85–2.83 (m, 4H, 2CH_2_), 1.52–1.51 (m, 4H, 2CH_2_), 1.34–1.33 (m, 2H, CH_2_) ppm. ^13^C-NMR (DMSO-*d*_*6*_): *δ *= 165.8 (C=O), 156.1 (C), 142.9 (C), 136.1 (C), 130.3 (C), 129.2 (CH), 126.8 (CH), 119.6 (CH), 112.8 (CH), 47.0 (CH_2_), 44.0 (CH_2_), 25.1 (CH_2_), 23.3 (CH_2_) ppm.

#### *N*-(4-(Morpholinosulfonyl)phenyl)-2-(phenylamino)acetamide (**5i**)

Beige crystals, yield (59%), m.p. 120–122 °C. FT-IR: 3330 (NH), 3203 (NH), 3051 (CH arom.), 2957 (CH aliph.), 2860 (CH aliph.), 1701 (C=O) cm^−1^. ^1^H-NMR (DMSO-*d*_*6*_): *δ* = 10.85 (s, 1H, NH), 10.50 (s, 1H, NH), 7.88 (d, *J* = 10.0 Hz, 2H, Ph-H), 7.72 (d, *J* = 10.0 Hz, 2H, Ph-H), 7.14–7.10 (m, 2H, Ph-H), 6.67–6.63 (m, 3H, Ph-H), 4.34 (s, 2H, CH_2_), 3.64–3.62 (m, 4H, 2CH_2_), 2.85–2.83 (m, 4H, 2CH_2_) ppm. ^13^C-NMR (DMSO-*d*_*6*_): *δ* = 165.9 (C=O), 146.3 (C), 143.3 (C), 129.5 (CH), 129.4 (CH), 129.2 (C), 119.6 (CH), 119.4 (CH), 113.2 (CH), 66.7 (CH_2_), 46.3 (CH_2_), 44.0 (CH_2_) ppm.

#### 2-(4-Methoxyphenylamino)-*N*-(4-(morpholinosulfonyl)phenyl)acetamide (**5j**)

Pale yellow crystals, yield (67%), m.p. 97–99 °C. FT-IR: 3400 (NH), 3332 (NH), 3099 (CH arom.), 2973 (CH aliph.), 2858 (CH aliph.), 1689 (C=O) cm^−1^. ^1^H-NMR (DMSO-*d*_*6*_): *δ* = 11.43 (s, 1H, NH), 10.93 (s, 1H, NH), 7.89 (d, *J* = 11.0 Hz, 2H, Ph-H), 7.71 (d, *J* = 11.0 Hz, 2H, Ph-H), 7.04 (d, *J* = 11.0 Hz, 2H, Ph-H), 6.90 (d, 2H, *J* = 11.0 Hz, Ph-H), 4.35 (s, 2H, CH_2_), 4.14 (s, 3H, CH_3_), 3.65–3.63 (m, 4H, 2CH_2_), 2.84–2.82 (m, 4H, 2CH_2_) ppm. ^13^C-NMR (DMSO-*d*_*6*_): *δ* = 165.6 (C=O), 142.8 (C), 141.7 (C), 129.7 (CH), 129.2 (C), 119.5 (CH), 115.2 (CH), 65.7 (CH_2_), 55.8 (CH_3_), 46.3 (CH_2_), 44.0 (CH_2_) ppm.

#### *N*-(4-(morpholinosulfonyl)phenyl)-2-(*p*-tolylamino)acetamide (**5k**)

Beige crystals, yield (69%), m.p. 86–88 °C. FT-IR: 3332 (NH), 3190 (NH), 3104 (CH arom.), 2973 (CH aliph.), 2859 (CH aliph.), 1698 (C=O) cm^−1^. ^1^H-NMR (DMSO-*d*_*6*_): *δ* = 10.86 (s, 1H, NH), 10.83 (s, 1H, NH), 7.87 (d, *J* = 10.5 Hz, 2H, Ph-H), 7.72 (d, *J* = 10.0 Hz, 2H, Ph-H), 7.26–7.00 (m, 4H, Ph-H), 4.33 (s, 2H, CH_2_), 3.62–3.60 (m, 4H, 2CH_2_), 2.85–2.83 (m, 4H, 2CH_2_), 2.34 (s, 3H, CH_3_) ppm. ^13^C-NMR (DMSO-*d*_*6*_): *δ* = 165.9 (C=O), 143.3 (C), 131.7 (C), 129.4 (CH), 129.6 (C), 119.6 (CH), 65.7 (CH_2_), 46.3 (CH_2_), 44.0 (CH_2_), 20.2 (CH_3_) ppm.

#### 2-(4-Chlorophenylamino)-*N*-(4-(morpholinosulfonyl)phenyl)acetamide (**5l**)

White crystals, yield (55%), m.p. 102–104 °C. FT-IR: 3339 (NH), 3191 (NH), 3055 (CH arom.), 2971 (CH aliph.), 2857 (CH aliph.), 1695 (C=O) cm^−1^. ^1^H-NMR (DMSO-*d*_*6*_): *δ* = 10.84 (s, 1H, NH), 10.52 (s, 1H, NH), 7.87 (d, *J* = 10.5 Hz, 2H, Ph-H), 7.71 (d, *J* = 10.5 Hz, 2H, Ph-H), 7.12 (d, *J* = 11.0 Hz, 2H, Ph-H), 6.61 (d, *J* = 11.0 Hz, 2H, Ph-H), 4.33 (s, 2H, CH_2_), 3.63–3.61 (m, 4H, 2CH_2_), 2.84–2.82 (m, 4H, 2CH_2_) ppm. ^13^C-NMR (DMSO-*d*_*6*_): *δ *= 165.9 (C = O), 147.9 (C), 143.4 (C), 143.3 (C), 129.5 (CH), 129.1 (C), 119.6 (CH), 114.2 (CH), 65.7 (CH_2_), 46.3 (CH_2_), 44.0 (CH_2_) ppm.

#### 2-(4-Bromophenylamino)-*N*-(4-(morpholinosulfonyl)phenyl)acetamide (**5m**)

Beige crystals, yield (51%), m.p. 126–128 °C. FT-IR: 3341 (NH), 3101 (NH), 3060 (CH arom.), 2970 (CH aliph.), 2854 (CH aliph.), 1700 (C=O) cm^−1^. ^1^H-NMR (DMSO-*d*_*6*_): *δ* = 10.87 (s, 1H, NH), 10.54 (s, 1H, NH), 7.86 (d, *J* = 11.0 Hz, 2H, Ph-H), 7.71 (d, *J* = 11.0 Hz, 2H, Ph-H), 7.21 (d, *J* = 11.0 Hz, 2H, Ph-H), 6.58 (d, *J* = 11.0 Hz, 2H, Ph-H), 4.34 (s, 2H, CH_2_), 3.96–3.94 (m, 4H, 2CH_2_), 2.84–2.82 (m, 4H, 2CH_2_) ppm. ^13^C-NMR (DMSO-*d*_*6*_): *δ* = 165.9 (C=O), 147.4 (C), 143.3 (C), 132.2 (C), 131.9 (CH), 129.4 (C), 129.1 (CH), 119.6 (CH), 114.8 (CH), 65.7 (CH_2_), 46.3 (CH_2_), 44.0 (CH_2_) ppm.

#### Ethyl 4-(2-(4-(morpholinosulfonyl)phenylamino)-2-oxoethylamino)benzoate (**5n**)

Beige crystals, yield (78%), m.p. 90–92 °C. FT-IR: 3426 (NH), 3346 (NH), 3040 (CH arom.), 2982 (CH aliph.), 2865 (CH aliph.), 1722 (C=O), 1685 (C=O) cm^−1^. ^1^H-NMR (DMSO-*d*_*6*_): *δ* = 10.84 (s, 1H, NH), 10.65 (s, 1H, NH), 7.87 (d, *J* = 10.5 Hz, 2H, Ph-H), 7.71 (d, *J* = 10.5 Hz, 2H, Ph-H), 7.65 (d, *J* = 10.5 Hz, 2H, Ph-H), 6.59 (d, *J* = 10.5 Hz, 2H, Ph-H), 4.33 (s, 2H, CH_2_), 4.19 (q, *J* = 8.5 Hz, 2H, CH_2_), 3.62–3.60 (m, 4H, 2CH_2_), 2.84–2.82 (m, 4H, 2CH_2_), 1.26 (t, *J* = 8.5 Hz, 3H, CH_3_) ppm. ^13^C-NMR (DMSO-*d*_*6*_): *δ* = 166.3 (C=O), 165.9 (C=O), 153.2 (C), 143.3 (C), 131.4 (CH), 129.4 (CH), 129.1 (C), 119.6 (CH), 116.9 (C), 113.4 (CH), 65.7 (CH_2_), 59.9 (CH_2_), 46.3 (CH_2_), 44.0 (CH_2_), 14.8 (CH_3_) ppm.

#### 4-(2-(4-(Morpholinosulfonyl)phenylamino)-2-oxoethylamino)benzoic acid (**5o**)

Beige crystals, yield (55%), m.p. 105–108 °C. FT-IR: 3475 (br, OH), 3365 (NH), 3059 (CH arom.), 2977 (CH aliph.), 2858 (CH aliph.), 1699 (C=O.), 1690 (C=O) cm^−1^. ^1^H-NMR (DMSO-*d*_*6*_): *δ* = 10.87 (s, 1H, NH), 10.71 (s, 1H, NH), 7.87 (d, *J* = 10.0 Hz, 2H, Ph-H), 7.73–7.69 (m, 4H, Ph-H), 6.66 (d, *J* = 10.5 Hz, 2H, Ph-H), 4.86 (s, 1H, OH), 4.33 (s, 2H, CH_2_), 3.63–3.61 (m, 4H, 2CH_2_), 2.84–2.82 (m, 4H, 2CH_2_) ppm. ^13^C-NMR (DMSO-*d*_*6*_): *δ* = 166.3 (C=O), 165.9 (C=O), 143.2 (C), 131.9 (CH), 129.5 (C), 129.4 (CH), 119.6 (C), 119.5 (CH), 113.8 (CH), 65.7 (CH_2_), 46.3 (CH_2_), 44.0 (CH_2_) ppm.

#### *N*-(4-(Morpholinosulfonyl)phenyl)-2-(4-nitrophenylamino)acetamide (**5p**)

Yellow crystals, yield (85%), m.p. 112–114 °C. FT-IR: 3343 (NH), 3362 (NH), 3055 (CH arom.), 2970 (CH aliph.), 2854 (CH aliph.), 1694 (C=O) cm^−1^. ^1^H-NMR (DMSO-*d*_*6*_): *δ* = 10.77 (s, 1H, NH), 7.94 (d, *J* = 11.0 Hz, 2H, Ph-H), 7.86 (d, *J* = 10.5 Hz, 2H, Ph-H), 7.71 (d, *J* = 10.5 Hz, 2H, Ph-H), 6.72 (s, 1H, NH), 6.60 (d, *J* = 11.0 Hz, 2H, Ph-H), 4.32 (s, 2H, CH_2_), 3.63-3.61 (m, 4H, 2CH_2_), 2.84–2.82 (m, 4H, 2CH_2_) ppm. ^13^C-NMR (DMSO-*d*_*6*_): *δ *= 165.9 (C=O), 156.1 (C), 143.3 (C), 136.1 (C), 129.4 (CH), 129.1 (C), 126.8 (CH), 119.6 (CH), 112.8 (CH), 65.7 (CH_2_), 46.3 (CH_2_), 44.0 (CH_2_) ppm.

### General procedure for synthesis of *N*-aryl-2-(4-(piperidin-1-ylsulfonyl)phenyl-amino)acetamides **6a**–**h** and *N*-aryl-2-(4-(morpholinosulfonyl)phenylamino)- acetamides **6i**–**p**

In a round bottomed flask, 2-chloro-*N*-arylacetamide **4a**–**h** (0.001 mol), sulfonamide **1a,b** (0.001 mol) and triethylamine (0.1 mL) in absolute ethanol (20 mL) was refluxed for 4-6 h. The reaction mixture was cooled to room temperature, the solid obtained was filtered, washed with cold ethanol, dried and recrystallized from ethanol to give the titled products **6a**–**p** and **6i**–**p**.

#### *N*-Phenyl-2-(4-(piperidin-1-ylsulfonyl)phenylamino)acetamide (**6a**)

White crystals, yield (96%), m.p. 122–124 °C. FT-IR: 3437 (NH), 3363 (NH), 3040 (CH arom.), 2950 (CH aliph.), 2836 (CH aliph.), 1674 (C=O) cm^−1^. ^1^H-NMR (DMSO-*d*_*6*_): *δ* = 10.33 (s, 1H, NH), 7.61 (d, *J* = 10.0 Hz, 2H, Ph-H), 7.36-7.31 (m, 4H, Ph-H), 7.10–7.07 (m, 1H, Ph-H), 6.67–6.65 (m, 2H, Ph-H), 6.00 (br s, 1H, NH), 4.27 (s, 2H, CH_2_), 2.77 (t, *J* = 6.5 Hz, 4H, 2CH_2_), 1.52–1.50 (m, 4H, 2CH_2_), 1.34-1.32 (m, 2H, CH_2_) ppm. ^13^C-NMR (DMSO-*d*_*6*_): *δ* = 165.1 (C=O), 153.4 (C), 138.9 (C), 129.8 (CH), 129.3 (CH), 124.3 (CH), 120.5 (C), 119.8 (CH), 113.2 (CH), 47.0 (CH_2_), 44.0 (CH_2_), 25.1 (CH_2_), 23.4 (CH_2_) ppm.

#### *N*-(4-Methoxyphenyl)-2-(4-(piperidin-1-ylsulfonyl)phenylamino)acetamide (**6b**)

White crystals, yield (92%), m.p. 120–121 °C. FT-IR: 3437 (NH), 3340 (NH), 3072 (CH arom.), 2952 (CH aliph.), 2836 (CH aliph.), 1664 (C=O) cm^−1^. ^1^H-NMR (DMSO-*d*_*6*_): *δ* = 10.17 (s, 1H, NH), 7.51 (d, *J* = 10.5 Hz, 2H, Ph-H), 7.34 (d, *J* = 10.5 Hz, 2H, Ph-H), 6.91 (d, *J* = 10.5 Hz, 2H, Ph-H), 6.65 (d, 2H, *J* = 10.5 Hz, Ph-H), 6.04 (s, 1H, NH), 4.22 (s, 2H, CH_2_), 3.73 (s, 3H, CH_3_), 2.78 (t, *J* = 5.5 Hz, 4H, 2CH_2_), 1.54–1.52 (m, 4H, 2CH_2_), 1.34–1.32 (m, 2H, CH_2_) ppm. ^13^C-NMR (DMSO-*d*_*6*_): *δ* = 164.6 (C=O), 156.0 (C), 153.5 (C), 132.0 (C), 129.9 (CH), 121.4 (CH), 120.4 (C), 114.4 (CH), 113.1 (CH), 55.6 (CH_3_), 47.0 (CH_2_), 44.0 (CH_2_), 25.1 (CH_2_), 23.5 (CH_2_) ppm.

#### 2-(4-(Piperidin-1-ylsulfonyl)phenylamino)-*N*-*p*-tolylacetamide (**6c**)

White crystals, yield (98%), m.p. 137–138 °C. FT-IR: 3437 (NH), 3363 (NH), 3040 (CH arom.), 2950 (CH aliph.), 2836 (CH aliph.), 1672 (C=O) cm^−1^. ^1^H-NMR (DMSO-*d*_*6*_): *δ* = 10.22 (s, 1H, NH), 7.48 (d, *J* = 10.5 Hz, 2H, Ph-H), 7.34 (d, *J* = 10.5 Hz, 2H, Ph-H), 7.13 (d, *J* = 10.5 Hz, 2H, Ph-H), 6.65 (d, 2H, *J* = 10.5 Hz, Ph-H), 6.04 (s, 1H, NH), 4.24 (s, 2H, CH_2_), 2.78 (t, *J* = 6.0 Hz, 4H, 2CH_2_), 2.26 (s, 3H, CH_3_), 1.53–1.51 (m, 4H, 2CH_2_), 1.34–1.32 (m, 2H, CH_2_) ppm. ^13^C-NMR (DMSO-*d*_*6*_): *δ* = 164.8 (C=O), 153.5 (C), 136.4 (C), 133.3 (C), 129.9 (CH), 129.7 (CH), 120.4 (C), 119.8 (CH), 113.1 (CH), 47.0 (CH_2_), 44.0 (CH_2_), 25.1 (CH_2_), 23.5 (CH_2_), 20.9 (CH_3_) ppm.

#### *N*-(4-Chlorophenyl)-2-(4-(piperidin-1-ylsulfonyl)phenylamino)acetamide (**6d**)

White crystals, yield (92%), m.p. 130–131 °C. FT-IR: 3437 (NH), 3362 (NH), 3040 (CH arom.), 2950 (CH aliph.), 2836 (CH aliph.), 1670 (C=O) cm^−1^. ^1^H-NMR (DMSO-*d*_*6*_): *δ* = 10.45 (s, 1H, NH), 7.63 (d, *J* = 10.5 Hz, 2H, Ph-H), 7.39 (d, *J* = 10.5 Hz, 2H, Ph-H), 7.33 (d, *J* = 10.5 Hz, 2H, Ph-H), 6.64 (d, 2H, *J* = 10.5 Hz, Ph-H), 6.04 (s, 1H, NH), 4.27 (s, 2H, CH_2_), 2.78 (t, *J* = 6.0 Hz, 4H, 2CH_2_), 1.53–1.51 (m, 4H, 2CH_2_), 1.34–1.32 (m, 2H, CH_2_) ppm. ^13^C-NMR (DMSO-*d*_*6*_): *δ* = 165.2 (C=O), 153.5 (C), 137.9 (C), 129.8 (CH), 129.2 (CH), 127.9 (C), 121.3 (CH), 120.4 (C), 113.1 (CH), 47.0 (CH_2_), 43.9 (CH_2_), 25.1 (CH_2_), 23.4 (CH_2_) ppm.

#### *N*-(4-Bromophenyl)-2-(4-(piperidin-1-ylsulfonyl)phenylamino)acetamide (**6e**)

White crystals, yield (97%), m.p. 145–146 °C. FT-IR: 3437 (NH), 3362 (NH), 3045 (CH arom.), 2950 (CH aliph.), 2836 (CH aliph.), 1671 (C=O) cm^−1^. ^1^H-NMR (DMSO-*d*_*6*_): *δ* = 10.45 (s, 1H, NH), 7.58 (d, *J* = 10.5 Hz, 2H, Ph-H), 7.51 (d, *J* = 10.5 Hz, 2H, Ph-H), 7.34 (d, *J* = 11.0 Hz, 2H, Ph-H), 6.65 (d, 2H, *J* = 11.0 Hz, Ph-H), 6.04 (s, 1H, NH), 4.27 (s, 2H, CH_2_), 2.78 (t, *J* = 6.5 Hz, 4H, 2CH_2_), 1.53–1.51 (m, 4H, 2CH_2_), 1.34–1.32 (m, 2H, CH_2_) ppm. ^13^C-NMR (DMSO-*d*_*6*_): *δ* = 165.2 (C=O), 153.4 (C), 138.3 (C), 132.1 (CH), 129.8 (CH), 121.7 (CH), 120.4 (C), 115.6 (C), 113.1 (CH), 47.0 (CH_2_), 44.0 (CH_2_), 25.1 (CH_2_), 23.4 (CH_2_) ppm.

#### Ethyl 4-(2-(4-(piperidin-1-ylsulfonyl)phenylamino)acetamido)benzoate (**6f**)

White crystals, yield (91%), m.p. 75–76 °C. FT-IR: 3275 (NH), 3203 (NH), 3090 (CH arom.), 2947 (CH aliph.), 2858 (CH aliph.), 1721 (C=O), 1679 (C=O) cm^−1^. ^1^H-NMR (DMSO-*d*_*6*_): *δ* = 10.75 (s, 1H, NH), 10.65 (s, 1H, NH), 7.94 (d, *J* = 10.5 Hz, 2H, Ph-H), 7.83 (d, *J* = 10.5 Hz, 2H, Ph-H), 7.74 (d, *J* = 10.5 Hz, 2H, Ph-H), 7.71 (d, 2H, *J* = 10.5 Hz, Ph-H), 4.29 (q, *J* = 6.0 Hz, 4H, 2CH_2_), 2.85 (t, *J* = 6.0 Hz, 4H, 2CH_2_), 1.54–1.52 (m, 4H, 2CH_2_), 1.34 (t, *J* = 6.0 Hz, 3H, CH_3_), 1.33–1.29 (m, 2H, CH_2_) ppm. ^13^C-NMR (DMSO-*d*_*6*_): *δ* = 165.8 (C=O), 165.6 (C=O), 143.2 (C), 142.9 (C), 130.8 (CH), 130.3 (C), 129.2 (CH), 125.6 (C), 119.6 (CH), 119.2 (CH), 60.9 (CH_2_), 47.0 (CH_2_), 44.0 (CH_2_), 25.1 (CH_2_), 23.3 (CH_2_), 14.6 (CH_3_) ppm.

#### 4-(2-(4-(Piperidin-1-ylsulfonyl)phenylamino)acetamido)benzoic acid (**6g**)

White crystals, yield (81%), m.p. 164–166 °C. FT-IR: 3437 (br OH), 3340 (NH), 3000 (CH arom.), 2950 (CH aliph.), 2836 (CH aliph.), 1680 (C=O), 1641 (C=O) cm^−1^. ^1^H-NMR (DMSO-*d*_*6*_): *δ* = 12.77 (s, 1H, OH), 10.94 (s, 1H, NH), 7.92 (d, *J* = 10.5 Hz, 2H, Ph-H), 7.76 (d, *J* = 10.5 Hz, 2H, Ph-H), 7.33 (d, *J* = 10.5 Hz, 2H, Ph-H), 6.65 (d, 2H, *J* = 10.5 Hz, Ph-H), 6.07 (s, 1H, NH), 4.35 (s, 2H, CH_2_), 2.79–2.76 (m, 4H, 2CH_2_), 1.53–1.50 (m, 4H, 2CH_2_), 1.35–1.33 (m, 2H, CH_2_) ppm. ^13^C-NMR (DMSO-*d*_*6*_): *δ* = 167.1 (C=O), 165.7 (C=O), 153.5 (C), 143.5 (C), 131.4 (CH), 129.8 (CH), 126.4 (C), 120.3 (C), 119.0 (CH), 113.1 (CH), 47.0 (CH_2_), 44.0 (CH_2_), 25.1 (CH_2_), 23.4 (CH_2_) ppm.

#### *N*-(4-Nitrophenyl)-2-(4-(piperidin-1-ylsulfonyl)phenylamino)acetamide (**6h**)

Yellow crystals, yield (85%), m.p. 142–144 °C. FT-IR: 3437 (NH), 3362 (NH), 3099 (CH arom.), 2947 (CH aliph.), 2836 (CH aliph.), 1685 (C=O) cm^−1^. ^1^H-NMR (DMSO-*d*_*6*_): *δ* = 10.92 (s, 1H, NH), 8.24 (d, *J* = 11.5 Hz, 2H, Ph-H), 7.85 (d, *J* = 11.5 Hz, 2H, Ph-H), 7.33 (d, *J* = 11.0 Hz, 2H, Ph-H), 6.65 (d, 2H, *J* = 11.0 Hz, Ph-H), 6.04 (s, 1H, NH), 4.35 (s, 2H, CH_2_), 2.77 (t, *J* = 6.0 Hz, 4H, 2CH_2_), 1.53–1.51 (m, 4H, 2CH_2_), 1.33–1.31 (m, 2H, CH_2_) ppm. ^13^C-NMR (DMSO-*d*_*6*_): *δ* = 166.0 (C=O), 153.4 (C), 145.0 (C), 143.0 (C), 129.8 (CH), 125.5 (CH), 120.4 (C), 119.5 (C), 113.1 (CH), 47.0 (CH_2_), 44.0 (CH_2_), 25.1 (CH_2_), 23.4 (CH_2_) ppm.

#### 2-(4-(Morpholinosulfonyl)phenylamino)-*N*-phenylacetamide (**6i**)

Beige crystals, yield (98%), m.p. 133–134 °C. FT-IR: 3444 (NH), 3365 (NH), 3099 (CH arom.), 2985 (CH aliph.), 2847 (CH aliph.), 1672 (C=O) cm^−1^. ^1^H-NMR (DMSO-*d*_*6*_): *δ* = 10.30 (s, 1H, NH), 7.59 (d, *J* = 9.5 Hz, 2H, Ph-H), 7.35 (d, *J* = 10.5 Hz, 2H, Ph-H), 7.32 (d, *J* = 9.5 Hz, 2H, Ph-H), 7.10–7.06 (m, 1H, Ph-H), 6.68–6.66 (m, 2H, Ph-H), 6.12 (s, 1H, NH), 4.26 (s, 2H, CH_2_), 3.62–3.60 (m, 4H, 2CH_2_), 2.77–2.75 (m, 4H, 2CH_2_) ppm. ^13^C-NMR (DMSO-*d*_*6*_): *δ* = 165.1 (C=O), 153.8 (C), 138.9 (C), 130.2 (CH), 129.3 (CH), 124.3 (CH), 119.8 (CH), 119.1 (C), 113.2 (CH), 65.7 (CH_2_), 46.4 (CH_2_), 44.0 (CH_2_) ppm.

#### *N*-(4-Methoxyphenyl)-2-(4-(morpholinosulfonyl)phenylamino)acetamide (**6j**)

Pale yellow crystals, yield (94%), m.p. 127–128 °C. FT-IR: 3444 (NH), 3364 (NH), 3073 (CH arom.), 2957 (CH aliph.), 2844 (CH aliph.), 1665 (C=O) cm^−1^. ^1^H-NMR (DMSO-*d*_*6*_): *δ* = 10.17 (s, 1H, NH), 7.51 (d, *J* = 11.0 Hz, 2H, Ph-H), 7.35 (d, *J* = 10.5 Hz, 2H, Ph-H), 6.90 (d, *J* = 11.0 Hz, 2H, Ph-H), 6.67 (d, *J* = 10.5 Hz, 2H, Ph-H), 6.13 (s, 1H, NH), 4.22 (s, 2H, CH_2_), 3.72 (s, 3H, CH_3_), 3.62–3.60 (m, 4H, 2CH_2_), 2.77–2.75 (m, 4H, 2CH_2_) ppm. ^13^C-NMR (DMSO-*d*_*6*_): *δ* = 164.6 (C=O), 156.0 (C), 153.8 (C), 132.0 (C), 130.2 (CH), 121.4 (CH), 119.1 (C), 114.4 (CH), 113.2 (CH), 65.7 (CH_2_), 55.6 (CH_3_), 46.3 (CH_2_), 44.0 (CH_2_), ppm.

#### 2-(4-(Morpholinosulfonyl)phenylamino)-*N*-*p*-tolylacetamide (**6k**)

Beige crystals, yield (55%), m.p. 158–160 °C. FT-IR: 3444 (NH), 3364 (NH), 3035 (CH arom.), 2916 (CH aliph.), 2848 (CH aliph.), 1673 (C=O) cm^−1^. ^1^H-NMR (DMSO-*d*_*6*_): *δ* = 10.21 (s, 1H, NH), 7.48 (d, *J* = 10.0 Hz, 2H, Ph-H), 7.36 (d, *J* = 10.5 Hz, 2H, Ph-H), 7.13 (d, *J* = 10.0 Hz, 2H, Ph-H), 6.68 (d, 2H, *J* = 10.5 Hz, Ph-H), 6.12 (s, 1H, NH), 4.23 (s, 2H, CH_2_), 3.61-3.59 (m, 4H, 2CH_2_), 2.77–2.75 (m, 4H, 2CH_2_), 2.25 (s, 3H, CH_3_) ppm. ^13^C-NMR (DMSO-*d*_*6*_): *δ* = 164.8 (C=O), 153.8 (C), 136.4 (C), 133.3 (C), 129.6 (CH), 129.8 (CH), 119.1 (CH), 113.2 (CH), 65.7 (CH_2_), 46.3 (CH_2_), 44.0 (CH_2_), 20.9 (CH_3_) ppm.

#### *N*-(4-Chlorophenyl)-2-(4-(morpholinosulfonyl)phenylamino)acetamide (**6l**)

White crystals, yield (98%), m.p. 166–168 °C. FT-IR: 3444 (NH), 3365 (NH), 3084 (CH arom.), 2984 (CH aliph.), 2847 (CH aliph.), 1670 (C=O) cm^−1^. ^1^H-NMR (DMSO-*d*_*6*_): *δ* = 10.44 (s, 1H, NH), 7.62 (d, *J* = 10.5 Hz, 2H, Ph-H), 7.39 (d, *J* = 10.5 Hz, 2H, Ph-H), 7.35 (d, *J* = 10.5 Hz, 2H, Ph-H), 6.67 (d, 2H, *J* = 10.5 Hz, Ph-H), 6.12 (s, 1H, NH), 4.26 (s, 2H, CH_2_), 3.61-3.59 (m, 4H, 2CH_2_), 2.77–2.75 (m, 4H, 2CH_2_) ppm. ^13^C-NMR (DMSO-*d*_*6*_): *δ* = 165.2 (C=O), 153.8 (C), 137.9 (C), 130.2 (CH), 129.2 (CH), 127.9 (C), 121.3 (CH), 119.1 (C), 113.2 (CH), 65.7 (CH_2_), 46.3 (CH_2_), 43.9 (CH_2_) ppm.

#### *N*-(4-Bromophenyl)-2-(4-(morpholinosulfonyl)phenylamino)acetamide (**6m**)

White crystals, yield (95%), m.p. 173–175 °C. FT-IR: 3444 (NH), 3365 (NH), 3078 (CH arom.), 2954 (CH aliph.), 2847 (CH aliph.), 1671 (C=O) cm^−1^. ^1^H-NMR (DMSO-*d*_*6*_): *δ* = 10.44 (s, 1H, NH), 7.57 (d, *J* = 10.5 Hz, 2H, Ph-H), 7.52 (d, *J* = 10.5 Hz, 2H, Ph-H), 7.35 (d, *J* = 10.5 Hz, 2H, Ph-H), 6.67 (d, 2H, *J* = 10.5 Hz, Ph-H), 6.12 (s, 1H, NH), 4.26 (s, 2H, CH_2_), 3.61-3.59 (m, 4H, 2CH_2_), 2.77–2.75 (m, 4H, 2CH_2_) ppm. ^13^C-NMR (DMSO-*d*_*6*_): *δ* = 165.2 (C=O), 153.8 (C), 138.3 (C), 132.1 (CH), 130.2 (CH), 121.7 (CH), 119.1 (C), 116.4 (C), 113.2 (CH), 65.7 (CH_2_), 46.4 (CH_2_), 44.0 (CH_2_) ppm.

#### Ethyl 4-(2-(4-(morpholinosulfonyl)phenylamino)acetamido)benzoate (**6n**)

White crystals, yield (94%), m.p. 97–99 °C. FT-IR: 3327 (NH), 3276 (NH), 3087 (CH arom.), 2971 (CH aliph.), 2863 (CH aliph.), 1720 (C=O), 1676 (C=O) cm^−1^. ^1^H-NMR (DMSO-*d*_*6*_): *δ* = 10.83 (s, 1H, NH), 10.69 (s, 1H, NH), 7.93 (d, *J* = 10.5 Hz, 2H, Ph-H), 7.87 (d, *J* = 10.5 Hz, 2H, Ph-H), 7.74 (d, *J* = 11.0 Hz, 2H, Ph-H), 7.71 (d, *J* = 11.0, 2H, Ph-H), 4.33–4.25 (m, 4H, 2CH_2_), 3.63–3.61 (m, 4H, 2CH_2_), 2.84–2.82 (m, 4H, 2CH_2_), 1.29 (t, *J* = 8.5 Hz, 3H, CH_3_) ppm. ^13^C-NMR (DMSO-*d*_*6*_): *δ* = 165.8 (C=O), 165.6 (C=O), 143.2 (C), 143.2 (C), 130.7 (CH), 129.4 (CH), 129.1 (C), 125.2 (C), 119.6 (CH), 119.2 (CH), 65.7 (CH_2_), 60.9 (CH_2_), 46.3 (CH_2_), 44.0 (CH_2_), 14.6 (CH_3_) ppm.

#### 4-(2-(4-(Morpholinosulfonyl)phenylamino)acetamido)benzoic acid (**6o**)

White crystals, yield (83%), m.p. 197–198 °C. FT-IR: 3444 (br OH), 3365 (NH), 3038 (CH arom.), 2984 (CH aliph.), 2847 (CH aliph.), 1682 (C=O),1642 (C=O) cm^−1^. ^1^H-NMR (DMSO-*d*_*6*_): *δ* = 12.12 (s, 1H, OH), 10.62 (s, 1H, NH), 7.92 (d, *J* = 10.5 Hz, 2H, Ph-H), 7.71 (d, *J* = 10.5 Hz, 2H, Ph-H), 7.35 (d, *J* = 10.5 Hz, 2H, Ph-H), 6.66 (d, 2H, *J* = 10.5 Hz, Ph-H), 6.12 (s, 1H, NH), 4.30 (s, 2H, CH_2_), 3.61–3.59 (m, 4H, 2CH_2_), 2.77–2.75 (m, 4H, 2CH_2_) ppm. ^13^C-NMR (DMSO-*d*_*6*_): *δ* = 167.3 (C=O), 165.7 (C=O), 153.8 (C), 142.8 (C), 131.0 (C), 130.9 (CH), 130.2 (CH), 126.4 (C), 119.1 (CH), 113.2 (CH), 65.7 (CH_2_), 46.3 (CH_2_), 44.0 (CH_2_) ppm.

#### 2-(4-(Morpholinosulfonyl)phenylamino)-*N*-(4-nitrophenyl)acetamide (**6p**)

Pale yellow crystals, yield (96%), m.p. 158–160 °C. FT-IR: 3444 (NH), 3365 (NH), 3097 (CH arom.), 2984 (CH aliph.), 2847 (CH aliph,), 1686 (C=O) cm^−1^. ^1^H-NMR (DMSO-*d*_*6*_): *δ* = 10.91 (s, 1H, NH), 8.24 (d, *J* = 10.5 Hz, 2H, Ph-H), 7.84 (d, *J* = 10.5 Hz, 2H, Ph-H), 7.35 (d, *J* = 10.0 Hz, 2H, Ph-H), 6.67 (d, 2H, *J* = 10.0 Hz, Ph-H), 6.11 (s, 1H, NH), 4.35 (s, 2H, CH_2_), 3.61–3.59 (m, 4H, 2CH_2_), 2.77–2.75 (m, 4H, 2CH_2_) ppm. ^13^C-NMR (DMSO-*d*_*6*_): *δ* = 166.0 (C=O), 153.8 (C), 145.0 (C), 143.0 (C), 130.1 (CH), 125.5 (CH), 119.5 (CH), 119.1 (C), 113.2 (CH), 65.7 (CH_2_), 46.3 (CH_2_), 44.0 (CH_2_) ppm.

### Antimicrobial screening

All microbial strains were provided from culture collection of the Regional Center for Mycology and Biotechnology (RCMB), Al-Azhar University, Cairo, Egypt. The antimicrobial activity was evaluated by disc-agar diffusion method [62–64] using Mueller–Hinton Agar. Activation of various strains of bacteria occurred by using a loop that full of bacterial strain in the broth and incubated for 24 h at 37 °C. Furthermore, 0.1 mL of the suspension of strains was poured on the agar, spread well and left to solidify. Moreover, using a sterile cork, about 0.9 cm cut was made and were filled completely with the tested compound solution. The wells were incubated at 37 °C for 24 h. Each assay was done in triplicate. ZOI was determined using the mean value. Tested compound giving high ZOI value reflecting its significant antibacterial activity. Efficacy of the novel compounds was measured against different strains of Gram positive and Gram-negative bacteria, also evaluated against different fungal strains. The Gram-positive organisms that were used for culture sensitivity include S. aureus *(*RCMB010010), and *B. subtilis* RCMB 015 (1) NRRL B-543, on the other hand, the Gram-negative organisms that were used for culture sensitivity include *E. coli* (RCMB 010052) ATCC 25955, and *P. vulgaris* RCMB 004 (1) ATCC 13315. The fungal strains that were used include A. fumigatus (RCMB 002008), and *C. albicans* RCMB 005003 (1) ATCC 10231. Different antibiotics were used as a reference for evaluating the antimicrobial activity of novel compounds. Gentamycin and Ketoconazole were used as a reference antibiotic for assessing the antimicrobial activity of the novel compounds against bacterial and fungal strains, respectively.

### In vitro anticancer screening

The cell lines were purchased from the American Type Culture collection and their accession number as follows: A-549 (ATCC CCL-185™) lung carcinoma cell line and MCF-7 (ATCC HTB-22™) breast adenocarcinoma cell line.

Cytotoxic activity screening was performed at Regional Center for Mycology and Biotechnology, Al-Azhar University, according to the suggested method of Skehan et al. [65]. Exponentially, cells were placed in 10^4^ cells/well for 24 h, and then add fresh medium which containing different concentration of the tested sample. Serial two-fold dilutions of the tested sample were added using a multichannel pipette. Moreover, all cells were cultivated at 37 °C, 5% CO_2_ and 95% humidity. Also, incubation of control cells occurred at 37 °C. However, after incubation for 24 h different concentrations of sample (500, 250, 125, 62.50, 31.25, 15.60, 7.80, 3.90, 2, 1 and 0 µg L^−1^) were added and continued the incubation for 48 h, then, add the crystal violet solution 1% to each well for 0.5 h to examine viable cells. Rinse the wells using water until no stain. After that, add 30% glacial acetic acid to all wells with shaking plates on Microplate reader (TECAN, Inc.) to measure the absorbance, using a test wavelength of 490 nm. Besides, compare the treated samples with the control cell. The cytotoxicity was estimated by IC_50_ in µM; the concentration that inhibits 50% of growth of cancer cell.

### Docking and molecular modeling calculations

Were done in the Department of Pharmaceutical Organic Chemistry, Faculty of Pharmacy (Girls), Al-Azhar University, Egypt, as computational software using Protein Data Bank, 5-Fluorouracil (PDB ID: 1BID) and DHFR (PDB ID: 4DFR).

## Additional file

**Additional file 1.** A molecular docking study was executed to identify their good binding interactions with the active sites of dihydrofolate reductase (DHFR). Most of compounds displayed significant anticancer activity against human lung carcinoma (A-549) and human breast carcinoma (MCF-7) cell lines.

## Data Availability

Supporting information with NMR spectra and molecular docking are attached.
