# Supplementary material for: Design, synthesis, and biological evaluation of novel N4-substituted sulfonamides: acetamides derivatives as dihydrofolate reductase (DHFR) inhibitors
Source: BMC Chem. 2019 Jul 11;13(1):91. doi: 10.1186/s13065-019-0603-x (PMC6661844; doi:10.1186/s13065-019-0603-x)
Supplement: Supplementary file 1 — Additional file 1. A molecular docking study was executed to identify their good binding interactions with the active sites of dihydrofolate reductase (DHFR). Most of compounds displayed significant anticancer activity against human lung carcinoma (A-549) and human breast carcinoma (MCF-7) cell lines. [file 13065_2019_603_MOESM1_ESM.doc]

# SUPPORTING INFORMATION FOR

**Design, synthesis, and biological evaluation of novel *N^4^*-substituted sulfonamides: Acetamides derivatives as dihydrofolate reductase (DHFR) inhibitors**

Essam M. Hussein^1,2^*****, Munirah M. Al-Rooqi^1^, Shimaa M. Abd El-Gilil^3^, Saleh A. Ahmed^1,2^*****

*^1^ Department of Chemistry, Faculty of Applied Science, Umm Al-Qura University, Saudi Arabia*

*^2^ Chemistry Department, Faculty of Science, Assiut University, Assiut, Egypt*

*^3^ Department of pharmaceutical organic chemistry, faculty of pharmacy (girls), Al-azhar University, Nacr City, Cairo, Egypt*

1. **Spectral data of the new synthesized compounds**

**^1^H NMR spectrum of 2-(phenylamino)-*N*-(4-(piperidin-1-ylsulfonyl)phenyl)-acetamide (5a)**

******

**^13^C NMR spectrum of 2-(phenylamino)-*N*-(4-(piperidin-1-ylsulfonyl)phenyl)acetamide (5a)**

**Dept-135 NMR spectrum of 2-(phenylamino)-*N*-(4-(piperidin-1-ylsulfonyl)phenyl)acetamide (5a)**

**^1^H NMR spectrum of *N*-(4-(piperidin-1-ylsulfonyl)phenyl)-2-(*p*-tolylamino)acetamide** (**5c**)

**^13^C NMR spectrum of *N*-(4-(piperidin-1-ylsulfonyl)phenyl)-2-(*p*-tolylamino)acetamide** (**5c**)

**Dept-135 NMR spectrum of *N*-(4-(piperidin-1-ylsulfonyl)phenyl)-2-(*p*-tolylamino)acetamide** (**5c**)

**^1^H NMR spectrum of 2-(4-chlorophenylamino)-*N*-(4-(piperidin-1-ylsulfonyl)phenyl)acetamide** (**5d**)

**^13^C NMR spectrum of 2-(4-chlorophenylamino)-*N*-(4-(piperidin-1-ylsulfonyl)-phenyl)acetamide** (**5d**)

**DEPT-135 NMR spectrum of 2-(4-chlorophenylamino)-*N*-(4-(piperidin-1-ylsulfonyl)phenyl)acetamide** (**5d**)

**^1^H NMR spectrum of ethyl 4-(2-oxo-2-(4-(piperidin-1-ylsulfonyl)phenylamino)ethylamino)benzoate** (**5f**)

**^13^C NMR spectrum of ethyl 4-(2-oxo-2-(4-(piperidin-1-ylsulfonyl)phenylamino)-ethylamino)benzoate** (**5f**)

**Dept-135 NMR spectrum of ethyl 4-(2-oxo-2-(4-(piperidin-1-ylsulfonyl)phenylamino)ethylamino)-benzoate** (**5f**)

******

**^1^H NMR spectrum 2-(4-nitrophenylamino)-*N*-(4-(piperidin-1-ylsulfonyl)phenyl)acetamide** (**5h**)

******

**^13^C NMR spectrum 2-(4-nitrophenylamino)-*N*-(4-(piperidin-1-ylsulfonyl)phenyl)acetamide** (**5h**)

******

**Dept-135 NMR spectrum 2-(4-nitrophenylamino)-*N*-(4-(piperidin-1-ylsulfonyl)phenyl)acetamide** (**5h**)

******

**^1^H-^13^C-COSY NMR spectrum 2-(4-nitrophenylamino)-*N*-(4-(piperidin-1-ylsulfonyl)phenyl)acetamide** (**5h**)

******

**^1^H NMR spectrum of ethyl 4-(2-(4-(morpholinosulfonyl)phenylamino)-2-oxoethylamino)benzoate** (**5n**)

******

**^13^C NMR spectrum of ethyl 4-(2-(4-(morpholinosulfonyl)phenylamino)-2-oxoethylamino)benzoate** (**5n**)

******

**Dept-135 NMR spectrum of ethyl 4-(2-(4-(morpholinosulfonyl)phenylamino)-2-oxoethylamino)-benzoate** (**5n**)

******

**^1^H NMR spectrum of *N*-(4-(morpholinosulfonyl)phenyl)-2-(4-nitrophenylamino)acetamide** (**5p**)

**^13^C NMR spectrum of *N*-(4-(morpholinosulfonyl)phenyl)-2-(4-nitrophenylamino)acetamide** (**5p**)

**Dept-135 NMR spectrum of *N*-(4-(morpholinosulfonyl)phenyl)-2-(4-nitrophenylamino)acetamide** (**5p**)

**^1^H NMR spectrum of *N*-phenyl-2-(4-(piperidin-1-ylsulfonyl)phenylamino)acetamide (6a)**

**^13^C NMR spectrum of *N*-phenyl-2-(4-(piperidin-1-ylsulfonyl)phenylamino)acetamide (6a)**

**Dept-135 NMR spectrum of *N*-phenyl-2-(4-(piperidin-1-ylsulfonyl)phenylamino)acetamide (6a)**

**^1^H NMR spectrum of *N*-(4-methoxyphenyl)-2-(4-(piperidin-1-ylsulfonyl)phenylamino)acetamide** (**6b**)

**^13^C NMR spectrum of *N*-(4-methoxyphenyl)-2-(4-(piperidin-1-ylsulfonyl)phenylamino)acetamide** (**6b**)

**Dept-135 NMR spectrum of *N*-(4-methoxyphenyl)-2-(4-(piperidin-1-ylsulfonyl)phenylamino)acetamide** (**6b**)

**^1^H NMR spectrum of 2-(4-(piperidin-1-ylsulfonyl)phenylamino)-*N*-*p*-tolylacetamide (6c)**

**^13^C NMR spectrum of 2-(4-(piperidin-1-ylsulfonyl)phenylamino)-*N*-*p*-tolylacetamide (6c)**

**Dept-135 NMR spectrum of 2-(4-(piperidin-1-ylsulfonyl)phenylamino)-*N*-*p*-tolylacetamide (6c)**

**^1^H NMR spectrum of *N*-(4-chlorophenyl)-2-(4-(piperidin-1-ylsulfonyl)phenylamino)acetamide (6d)**

**^13^C NMR spectrum of *N*-(4-chlorophenyl)-2-(4-(piperidin-1-ylsulfonyl)phenylamino)acetamide (6d)**

**Dept-135 NMR spectrum of *N*-(4-chlorophenyl)-2-(4-(piperidin-1-ylsulfonyl)phenylamino)acetamide (6d)**

**^1^H NMR spectrum of *N*-(4-bromophenyl)-2-(4-(piperidin-1-ylsulfonyl)phenylamino)acetamide (6e)**

**^13^C NMR spectrum of *N*-(4-bromophenyl)-2-(4-(piperidin-1-ylsulfonyl)phenylamino)acetamide (6e)**

**Dept-135 NMR spectrum of *N*-(4-bromophenyl)-2-(4-(piperidin-1-ylsulfonyl)phenylamino)acetamide (6e)**

**^1^H NMR spectrum of ethyl 4-(2-(4-(piperidin-1-ylsulfonyl)phenylamino)acetamido)benzoate (6f)**

**^13^C NMR spectrum of ethyl 4-(2-(4-(piperidin-1-ylsulfonyl)phenylamino)acetamido)benzoate (6f)**

**Dept-135 NMR spectrum of ethyl 4-(2-(4-(piperidin-1-ylsulfonyl)phenylamino)acetamido)-benzoate (6f)**

**^1^H NMR spectrum of *N*-(4-nitrophenyl)-2-(4-(piperidin-1-ylsulfonyl)phenylamino)acetamide (6h)**

**^13^C NMR spectrum of *N*-(4-nitrophenyl)-2-(4-(piperidin-1-ylsulfonyl)phenylamino)acetamide (6h)**

**Dept-135 NMR spectrum of *N*-(4-nitrophenyl)-2-(4-(piperidin-1-ylsulfonyl)phenylamino)-acetamide (6h)**

**^1^H NMR spectrum of 2-(4-(morpholinosulfonyl)phenylamino)-*N*-phenylacetamide (6i)**

**^13^C NMR spectrum of 2-(4-(morpholinosulfonyl)phenylamino)-*N*-phenylacetamide (6i)**

**Dept-135 NMR spectrum of 2-(4-(morpholinosulfonyl)phenylamino)-*N*-phenylacetamide (6i)**

**^1^H NMR spectrum of *N*-(4-methoxyphenyl)-2-(4-(morpholinosulfonyl)phenylamino)acetamide (6j)**

**13C NMR spectrum of *N*-(4-methoxyphenyl)-2-(4-(morpholinosulfonyl)phenylamino)acetamide (6j)**

**Dept-135 NMR spectrum of *N*-(4-methoxyphenyl)-2-(4-(morpholinosulfonyl)phenylamino)acetamide (6j)**

**^1^H NMR spectrum of 2-(4-(morpholinosulfonyl)phenylamino)-*N*-*p*-tolylacetamide (6k)**

**^13^C NMR spectrum of 2-(4-(morpholinosulfonyl)phenylamino)-*N*-*p*-tolylacetamide (6k)**

**Dept-135 NMR spectrum of 2-(4-(morpholinosulfonyl)phenylamino)-*N*-*p*-tolylacetamide (6k)**

**^1^H NMR spectrum of *N*-(4-chlorophenyl)-2-(4-(morpholinosulfonyl)phenylamino)acetamide (6l)**

**^13^C NMR spectrum of *N*-(4-chlorophenyl)-2-(4-(morpholinosulfonyl)phenylamino)acetamide (6l)**

**Dept-135 NMR spectrum of *N*-(4-chlorophenyl)-2-(4-(morpholinosulfonyl)phenylamino)acetamide (6l)**

**^1^H NMR spectrum of *N*-(4-bromophenyl)-2-(4-(morpholinosulfonyl)phenylamino)acetamide (6m)**

**^13^C NMR spectrum of *N*-(4-bromophenyl)-2-(4-(morpholinosulfonyl)phenylamino)acetamide (6m)**

**Dept-135 NMR spectrum of *N*-(4-bromophenyl)-2-(4-(morpholinosulfonyl)phenylamino)acetamide (6m)**

**^1^H NMR spectrum of ethyl 4-(2-(4-(morpholinosulfonyl)phenylamino)acetamido)benzoate (6n)**

**^13^C NMR spectrum of ethyl 4-(2-(4-(morpholinosulfonyl)phenylamino)acetamido)benzoate (6n)**

**Dept-135 NMR spectrum of ethyl 4-(2-(4-(morpholinosulfonyl)phenylamino)acetamido)benzoate (6n)**

**^1^H NMR spectrum of 2-(4-(morpholinosulfonyl)phenylamino)-*N*-(4-nitrophenyl)acetamide (6p)**

**^13^C NMR spectrum of 2-(4-(morpholinosulfonyl)phenylamino)-*N*-(4-nitrophenyl)acetamide (6p)**

**Dept-135 NMR spectrum of 2-(4-(morpholinosulfonyl)phenylamino)-*N*-(4-nitrophenyl)acetamide (6p)**

1. **Docking and Molecular Modeling Calculations**

**Materials**

Docking and molecular modeling calculations were carried out in the Department of Pharmaceutical Organic Chemistry, Faculty of Pharmacy (Girls), Al‐Azhar University. All the molecular studies were carried out on an Intel(R) Core(TM) i7-3632QM 2.20 *GHz* processor, 8.00 GB memory with windows 7 Ultimate operating system using Molecular Operating Environment (MOE 2015.10; Chemical Computing Group, Montreal, Canada) as the computational software. All the minimizations were performed with MOE until a RMSD gradient of 0.05 K Cal/mol∙Å with MMFF94X force field and the partial charges were automatically calculated.

**General Methodology**

The coordinates of the X-ray crystal structure of 5-Fluorouracil (5-FU) bound to dihydrofolate reductase (DHFR) enzyme (PDB ID: 4DFR) were obtained from Protein Data Bank (PDB ID: 1BID). Enzyme structures were checked for missing atoms, bonds and contacts. Hydrogen atoms were added to the enzyme structure. Water molecules and bound ligands were manually deleted. The ligand molecules were constructed using the builder molecule and were energy minimized. The active site was generated using the MOE-Alpha site finder. Dummy atoms were created from the obtained alpha spheres. Ligands were docked within the dihydrofolate reductase active sites using the MOE-Dock with simulated annealing used as the search protocol and MMFF94X molecular mechanics force field for 8000 interactions. The lowest energy conformation selected and subjected to an energy minimization using MMFF94X force field.

**Docking on the Active Site of Dihydrofolate Reductase (DHFR)**

The recent determination of the three dimensional co-crystal structure of dihydrofolate reductase complexed with the potent inhibitor, 5-Fluorouracil (5-FU) (PDB ID: 4DFR) has led to the development of a model for the topography of the binding site of dihydrofolate reductase.

**Docking and Molecular Modeling**

Thymidylate synthase and dihydrofolate reductase are among the main targets involved in anticancer and antimicrobial activity [1,2]. Molecular modeling study using Molecular Operating Environment (MOE) [3] module was performed in order to rationalize the observed anticancer activity of the newly synthesized compounds. Molecular docking studies further help in understanding the mode of action of the compounds through their various interactions with the active sites of dihydrofolate reductase.

**Docking simulation study of the new synthesized compounds.**

MOE docking studies of the inhibitors were performed using dihydrofolate reductase co-crystallized with methotrexate (PDB ID: 4DFR) as a template.

**Docking of compound 5a into DHFR**

The active site revealed the presence of two hydrogen bond interactions as nitrogen atom acted as a hydrogen bond donor and acceptor with amino acid residues Trp 22 (2.76 Å) and Leu 24 (3.37 Å) with energy -1.6 and 2.9 kcal/mol; respectively. This beside many hydrophobic interactions with various amino acid residues: Pro 25, Asn 23, Leu 24, Trp 22, Ser 49, Leu 28, Ala 19, Arg 52, as shown in (**Figure S1**).

**
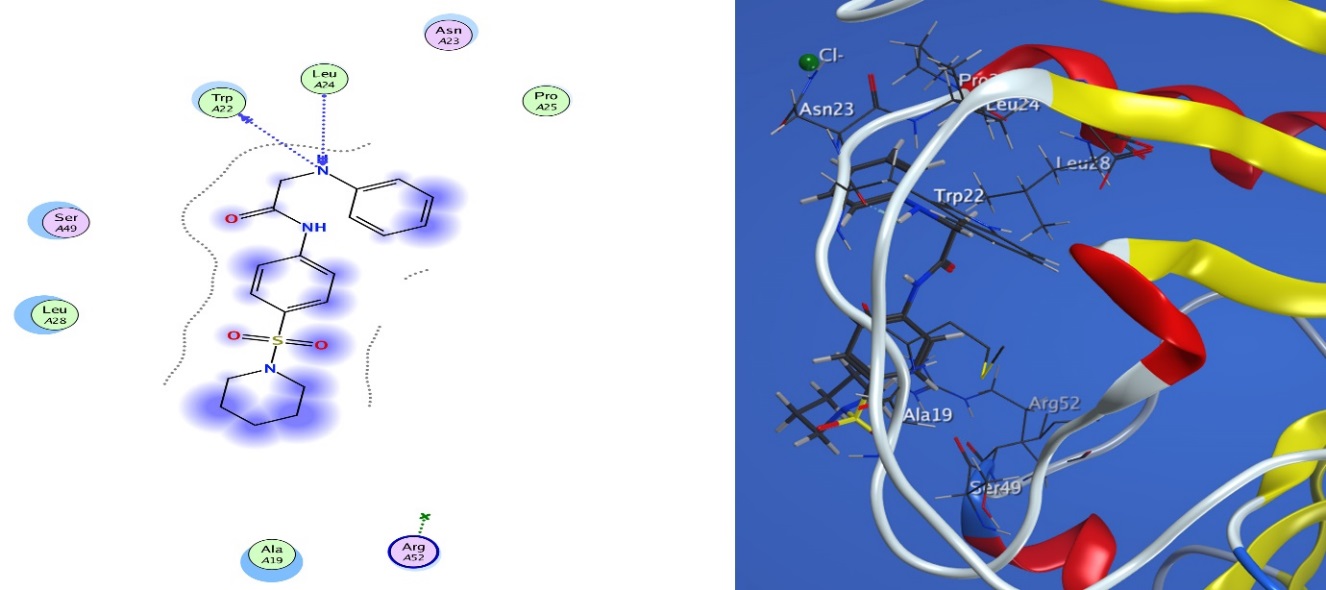
**

**Figure S1**. Docking of compound **5a** into DHFR

**Docking of compound 5b into DHFR**

The active site revealed the presence of an arene interaction between the phenyl ring and amino acid residue Asn 23 (4.22 Å) with energy -0.8 kcal/mol. This beside many hydrophobic interactions with various amino acid residues: Ser 49, Ala 19, Trp 22, Ile 50, Pro 25, Leu 28, Leu 24, Met 20, Arg 52, as shown in (**Figure S2**).

**
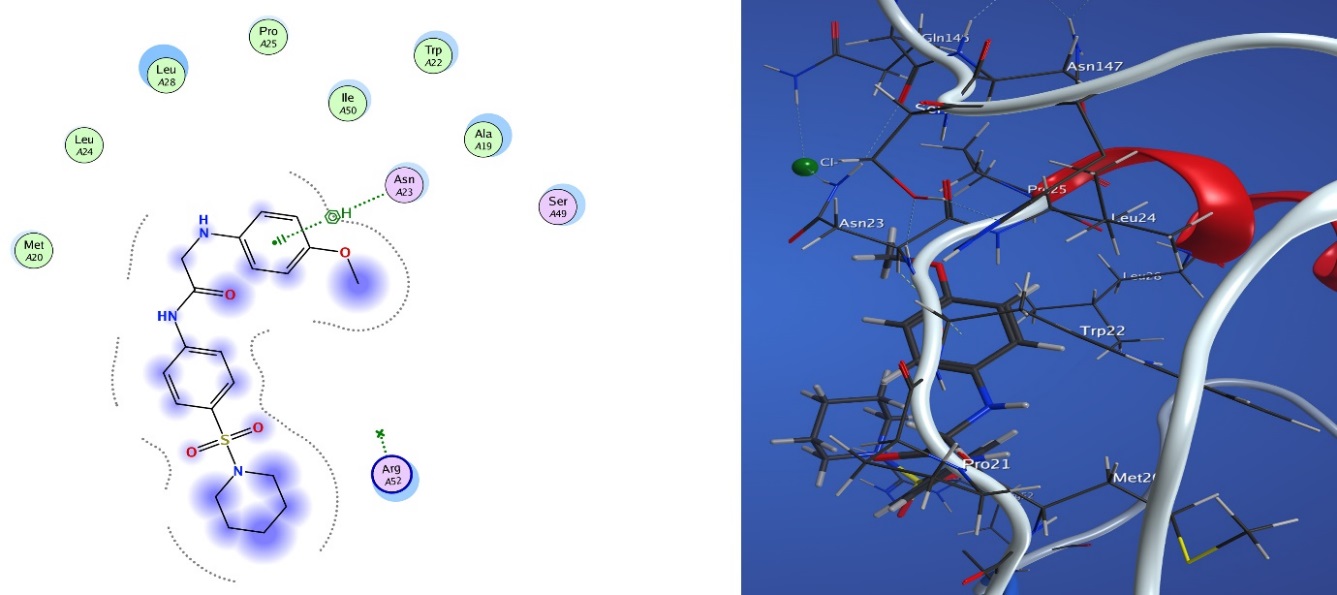
**

**Figure S2**. Docking of compound **5b** into DHFR

**Docking of compound 5c into DHFR**

The active site revealed that almost all atoms make hydrophobic interactions between many amino acid residues: Ala 145, Pro 21, Trp 22, Asp 144, Leu 28, Met 20, His 149, Asn 147, Asn 23, Ser 148, Gln 146, Arg 52, Ala 19, as shown in (**Figure S3**).


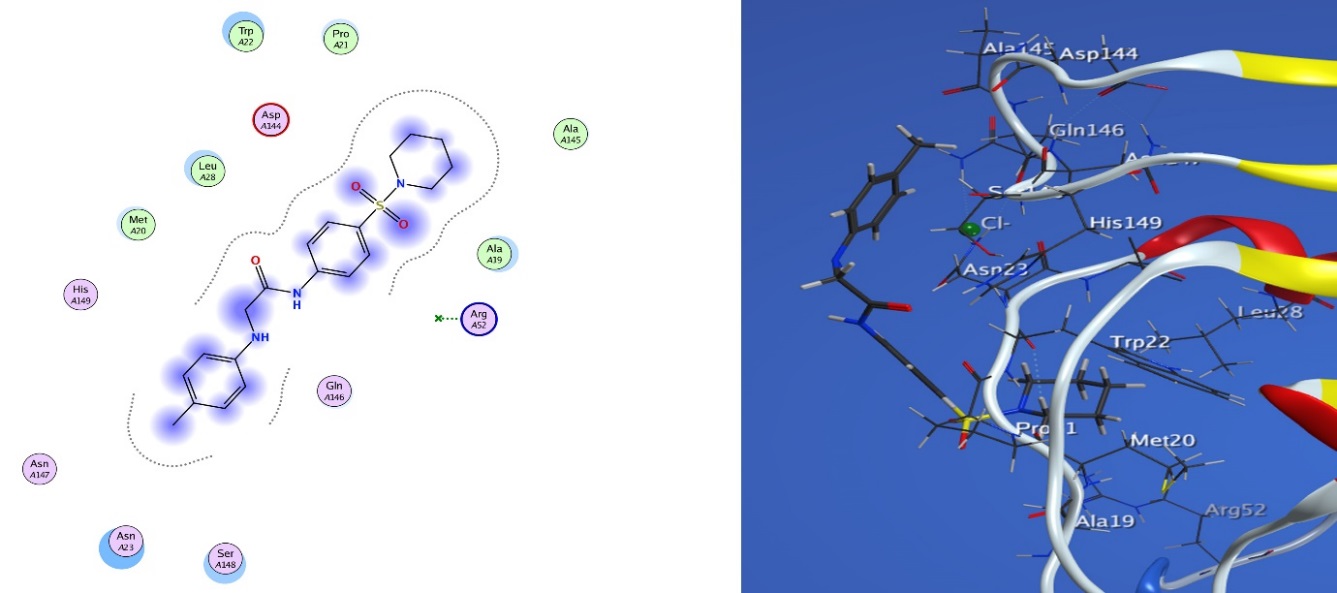


**Figure S3**. Docking of compound **5c** into DHFR

**Docking of compound 5d into DHFR**

The active site revealed the presence of one hydrogen bond interaction as chlorine atom acted as a hydrogen bond donor with amino acid residue Asp 144 (3.23 Å) with energy -1.9 kcal/mol. This beside many hydrophobic interactions with various amino acid residues: Asn 23, Asp 144, Ser 148, Asn 147, Arg 52, Ala 19, Ala 145, Pro 21, Gln 146, Leu 28, Trp 22, Leu 24, Met 20, as shown in (**Figure S4**).


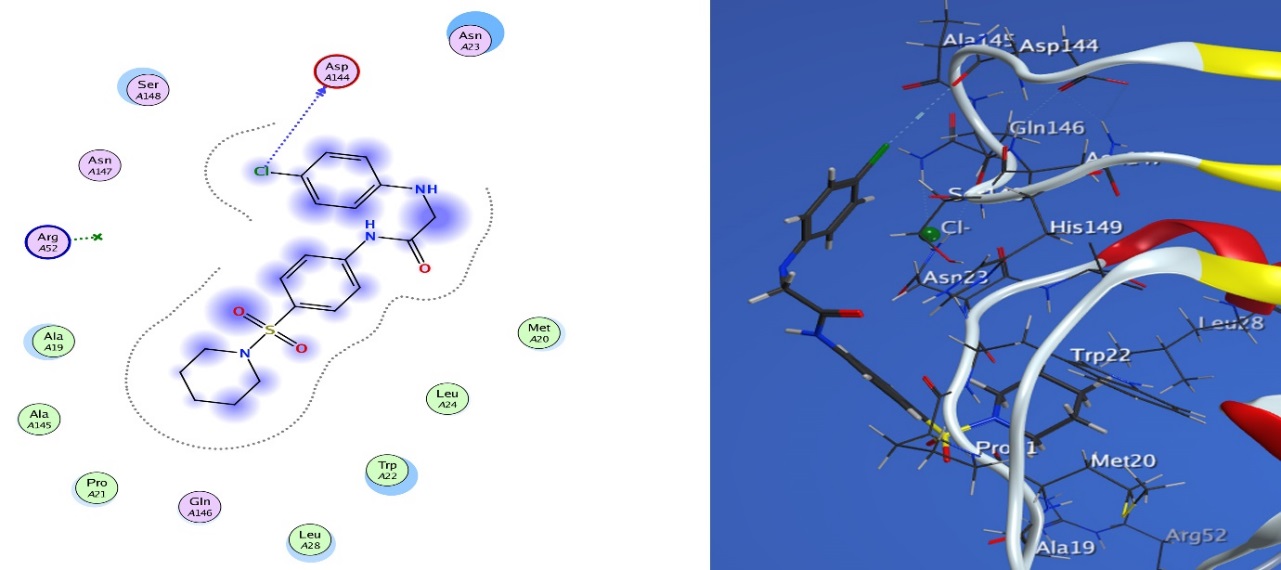


**Figure S4**. Docking of compound **5d** into DHFR

**Docking of compound 5e into DHFR**

The active site revealed the presence of one hydrogen bond interaction as bromine atom acted as a hydrogen bond donor with amino acid residue Asp 144 (3.64 Å) with energy -2.0 kcal/mol. This beside many hydrophobic interactions with various amino acid residues: Arg 52, Met 20, Asn 147, Trp 22, Leu 24, Asp 144, Pro 25, Ser 148, Pro 21, Ala 145, Gln 146, Leu 28, Asn 23, as shown in (**Figure S5**).


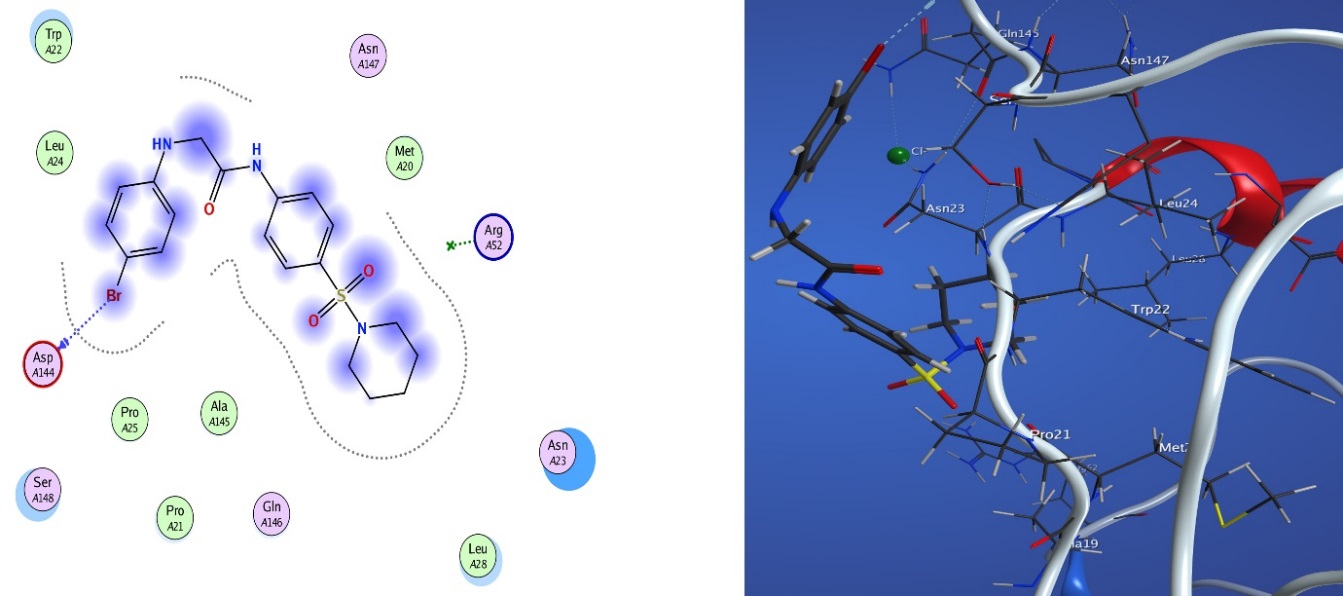


**Figure S5**. Docking of compound **5e** into DHFR

**Docking of compound 5f into DHFR**

The active site revealed that almost all atoms make hydrophobic interactions between many amino acid residues: Ser 148, Asn 147, Leu 24, Pro 21, Ala 19, Arg 52, Asp 144, Asn 23, Ala 145, Leu 23, Pro 25, Met 20, Trp 22, Gln 146, as shown in (**Figure S6**).

**
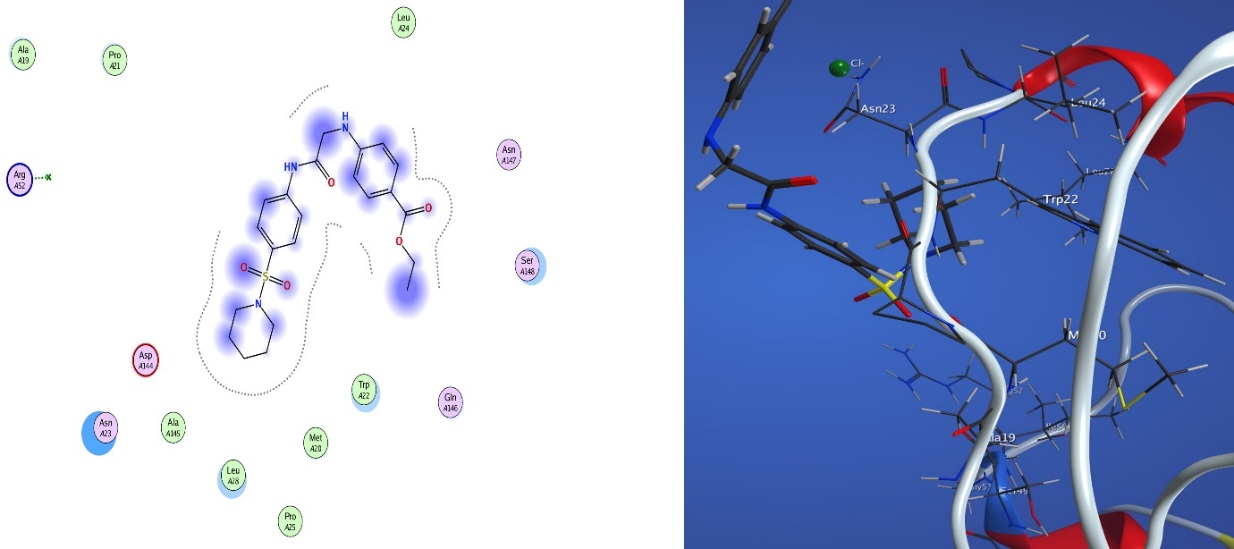
**

**Figure S6**. Docking of compound **5f** into DHFR

**Docking of compound 5h into DHFR**

The active site revealed that almost all atoms make hydrophobic interactions between many amino acid residues: Asn 23, Leu 28, Ser 49, Pro 25, Trp 22, Glu 48, Leu 24, Ile 50 Gly 51, Ala 19, Arg 52, as shown in (**Figure S7**).

**
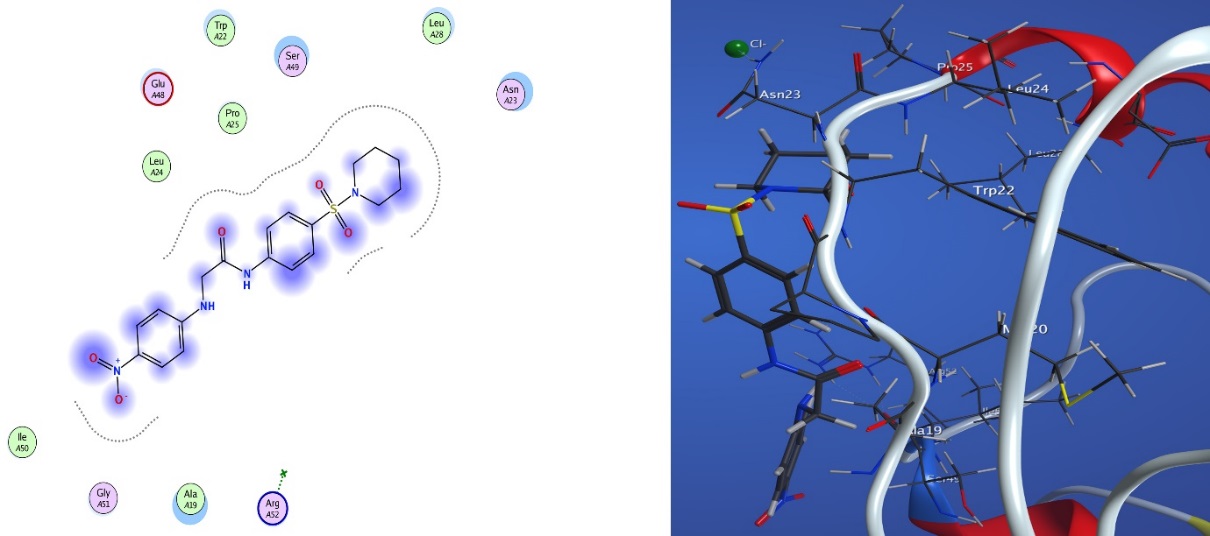
**

**Figure S7**. Docking of compound **5h** into DHFR

**Docking of compound 5i into DHFR**

The active site revealed that almost all atoms make hydrophobic interactions between many amino acid residues: Met 20, Leu 24, Leu 28, Trp 22, Pro 21, Ser 148, Arg 52, Ala 19, Asn 23, as shown in (**Figure S8**).

**
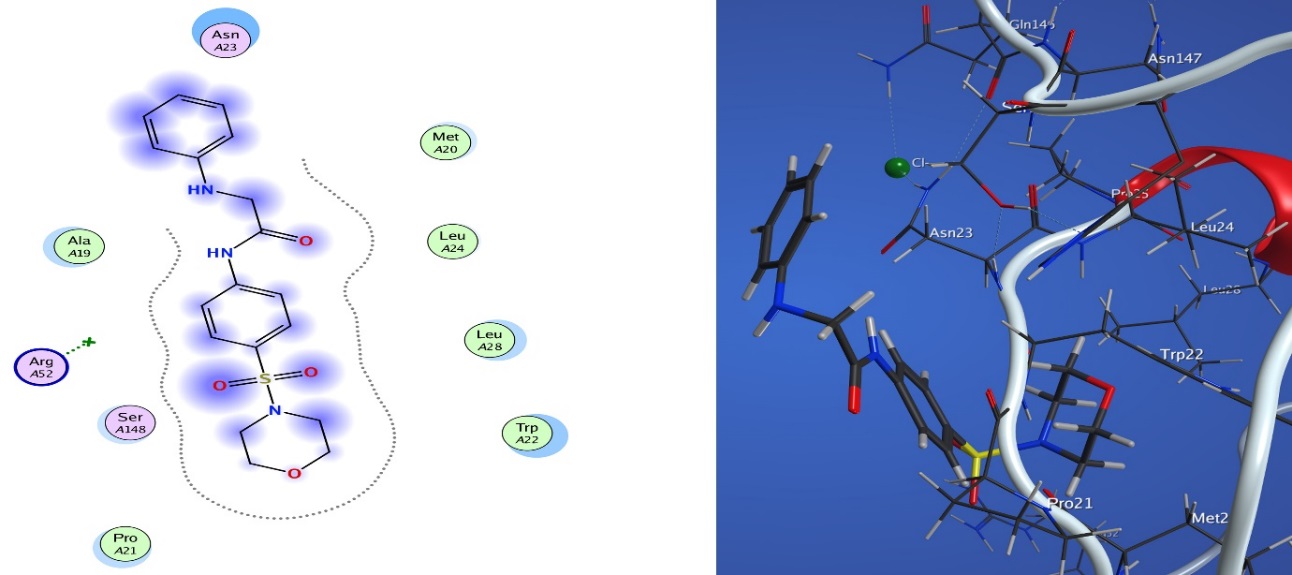
**

**Figure S8**. Docking of compound **5i** into DHFR

**Docking of compound 5k into DHFR**

The active site revealed that almost all atoms make hydrophobic interactions between many amino acid residues: Ala 19, Arg 52, Asn 23, Asn 147, Gln 146, Ser 148, Met 20, Asp 144, Ala 145, Trp 22, Pro 21, as shown in (**Figure S9**).

**
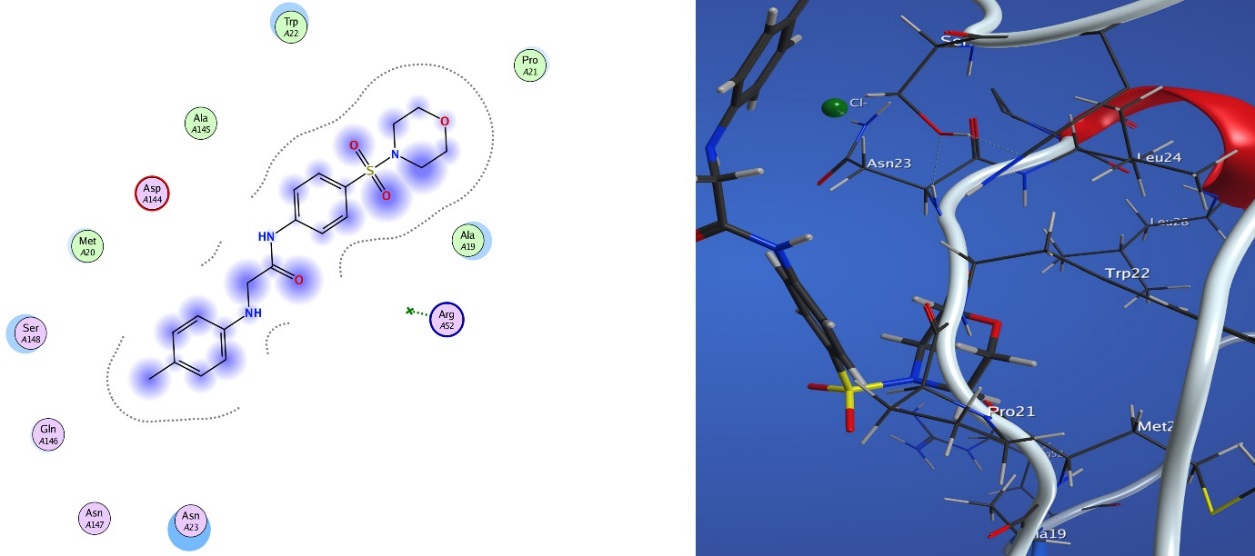
**

**Figure S9**. Docking of compound **5k** into DHFR

**Docking of compound 5l into DHFR**

The active site revealed that almost all atoms make hydrophobic interactions between many amino acid residues: Ile 50, Trp 22, Arg 52, Ala 19, Pro 25, Asn 23, Ser 49, Leu 24, Leu 28, Met 20, as shown in (**Figure S10**).

**
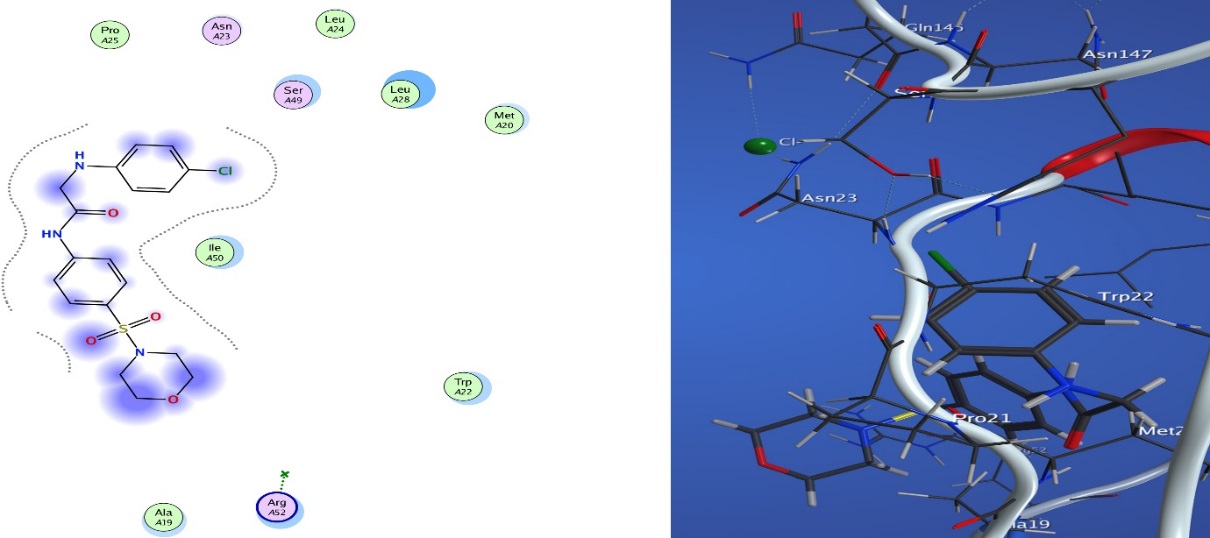
**

**Figure S10**. Docking of compound **5l** into DHFR

**Docking of compound 5m into DHFR**

The active site revealed that almost all atoms make hydrophobic interactions between many amino acid residues: Trp 22, Arg 52, Ala 19, Pro 25, Asn 23, Ser 49, Ile 50, Leu 28, Met 20, Leu 24, as shown in (**Figure S11**).

**
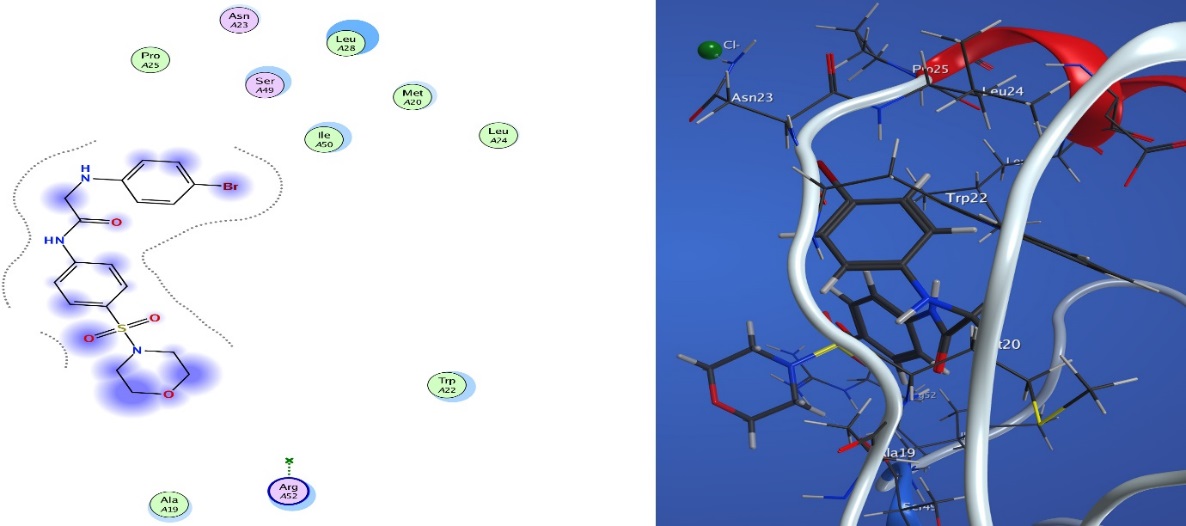
**

**Figure S11**. Docking of compound **5m** into DHFR

**Docking of compound 5n into DHFR**

The active site revealed that almost all atoms make hydrophobic interactions between many amino acid residues: Arg 52, Arg 71, Ala 19, Ile 50, Gly 51, Trp 22, Pro 25, Ser 49, Met 20, Asn 23, Leu 28, Leu 24, Glu 48, as shown in (**Figure S12**).

**
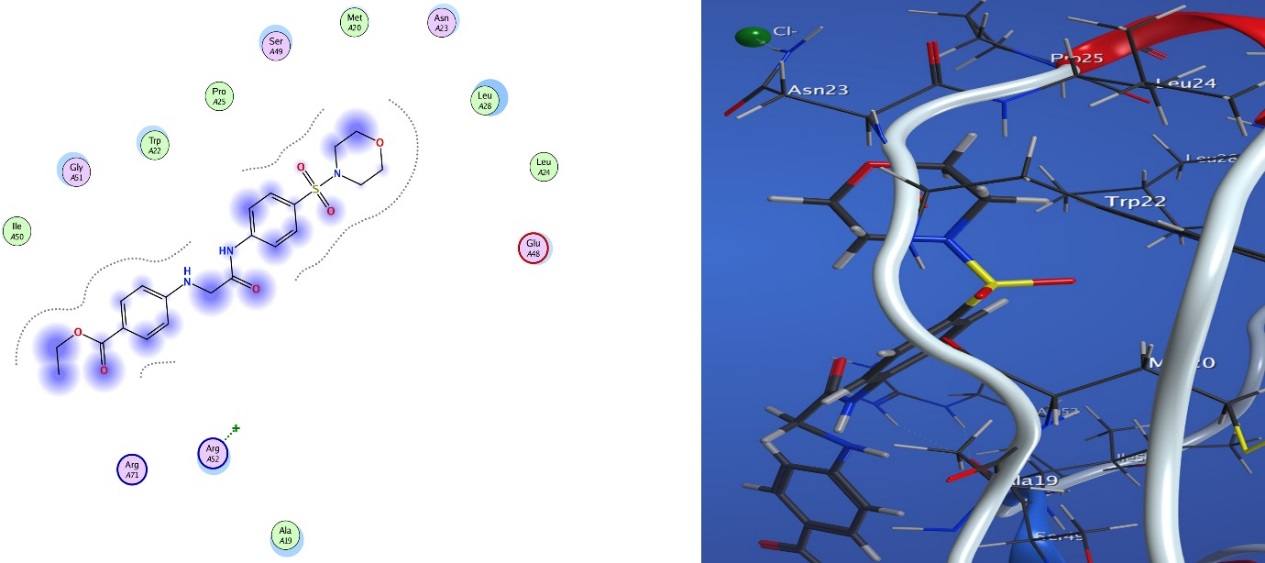
**

**Figure S12**. Docking of compound **5n** into DHFR

**Docking of compound 5o into DHFR**

The active site revealed the presence of one hydrogen bond interaction as oxygen atom of hydroxyl moiety acted as a hydrogen bond acceptor with amino acid residue Met 20 (3.11 Å) with energy -3.6 kcal/mol. This beside many hydrophobic interactions with various amino acid residues: Ile 50, Leu 24, Arg 52, Ala 19, Leu 28, Pro 25, Ser 49, Asn 23, Trp 22, as shown in (**Figure S13**).

**
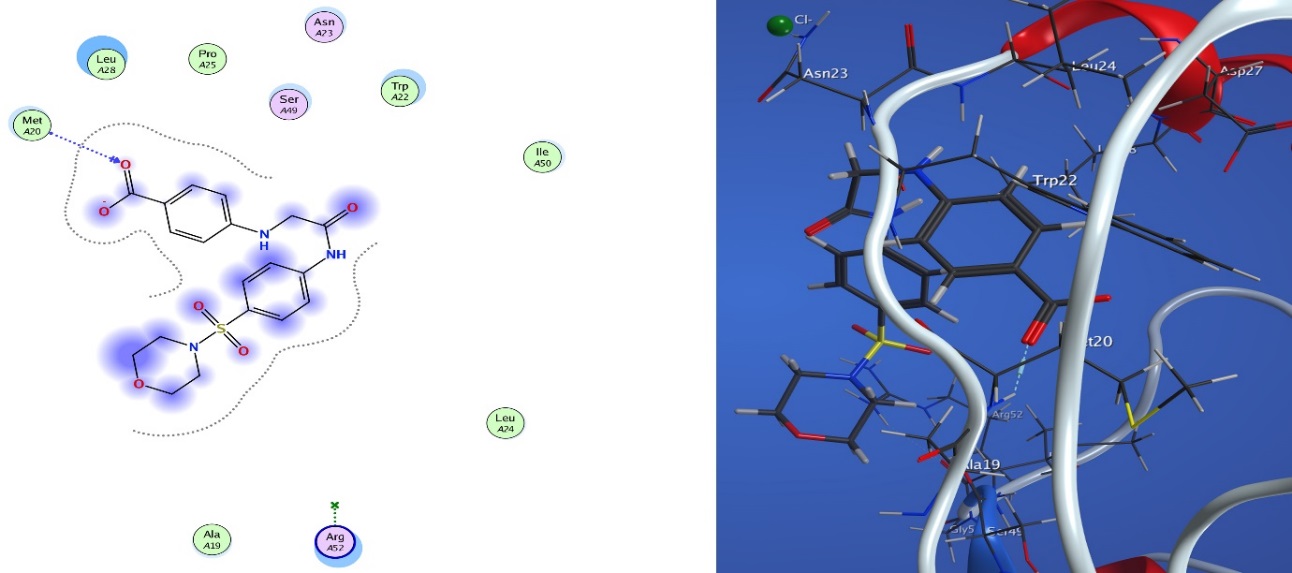
**

**Figure S13**. Docking of compound **5o** into DHFR

**Docking of compound 5p into DHFR**

The active site revealed the presence of an arene interaction between the phenyl ring and amino acid residue Asn 23 (3.94 Å) with energy -0.8 kcal/mol. This beside many hydrophobic interactions with various amino acid residues: Arg 52, Gly 51, Met 20, Leu 24, Pro 25, Ile 50, Leu 28, Ala 19, Glu 48, Trp 22, Ser 49, as shown in (**Figure S14**).

**
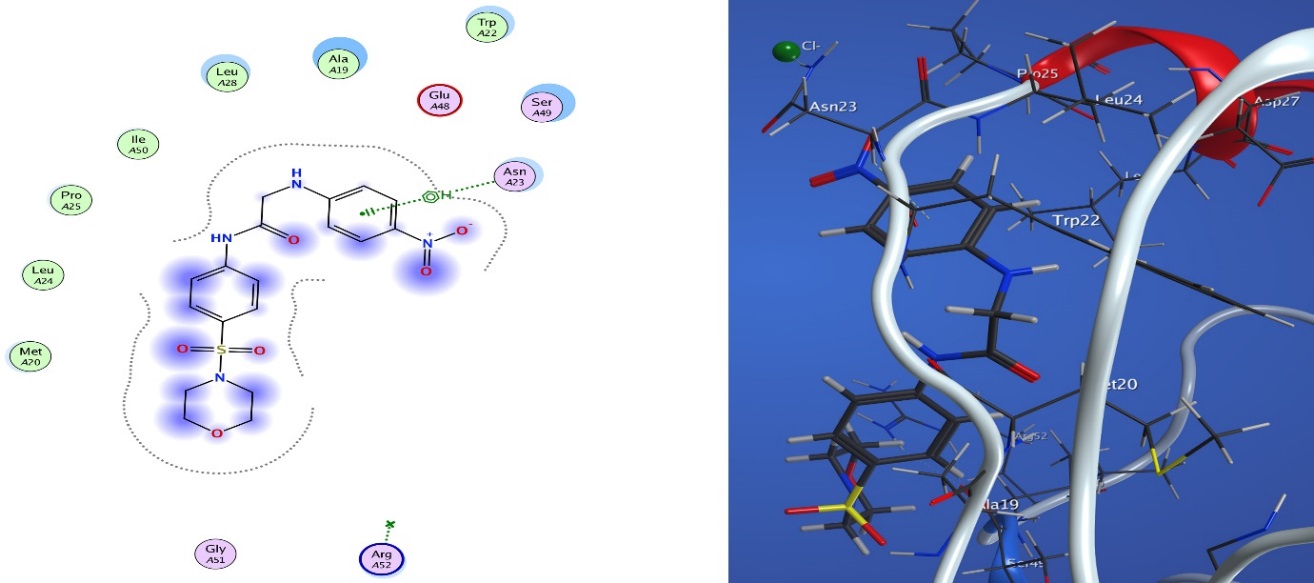
**

**Figure S14**. Docking of compound **5p** into DHFR

**Docking of compound 6a into DHFR**

The active site revealed that almost all atoms make hydrophobic interactions between many amino acid residues: Arg 52, Met 20, Trp 22, Leu 24, Gln 146, Ser 148, Asn 147, Leu 28 Pro 21, Asn 23, Ala 145, Asp 144, as shown in (**Figure S15**).

**
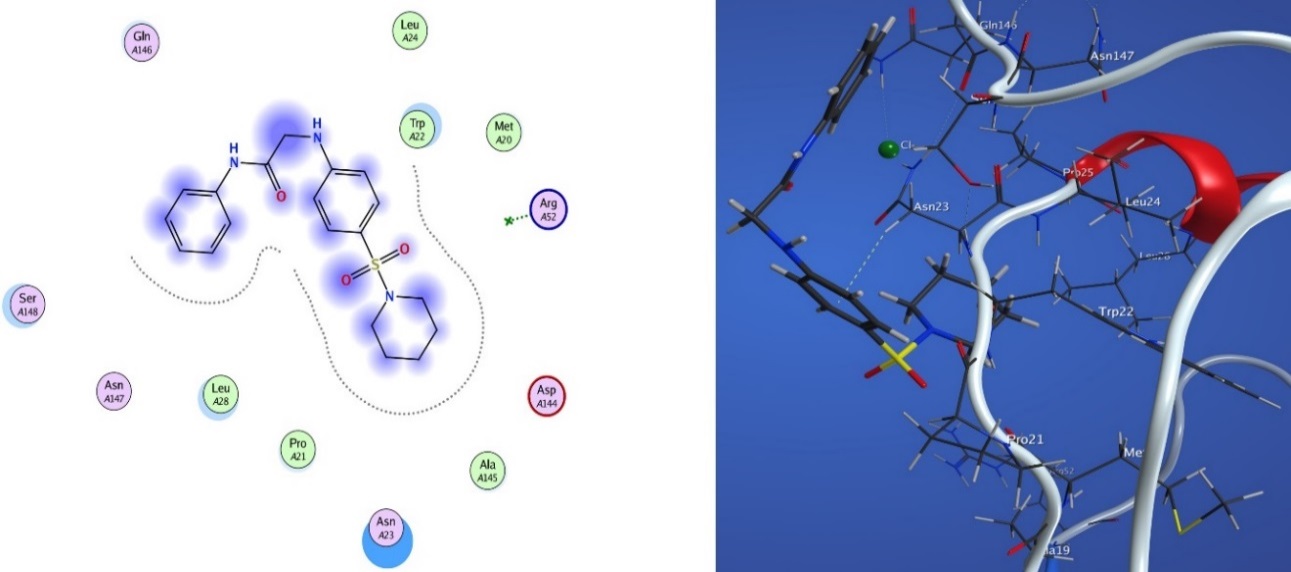
**

**Figure S15**. Docking of compound **6a** into DHFR

**Docking of compound 6b into DHFR**

The active site revealed that almost all atoms make hydrophobic interactions between many amino acid residues: Asn 147, Gln 146, Asp 144, Pro 21, Leu 28, Pro 25, Asn 23, Ala 145, Arg 52, Met 20, Leu 24, Trp 22, Ser 148, as shown in (**Figure S16**).

**
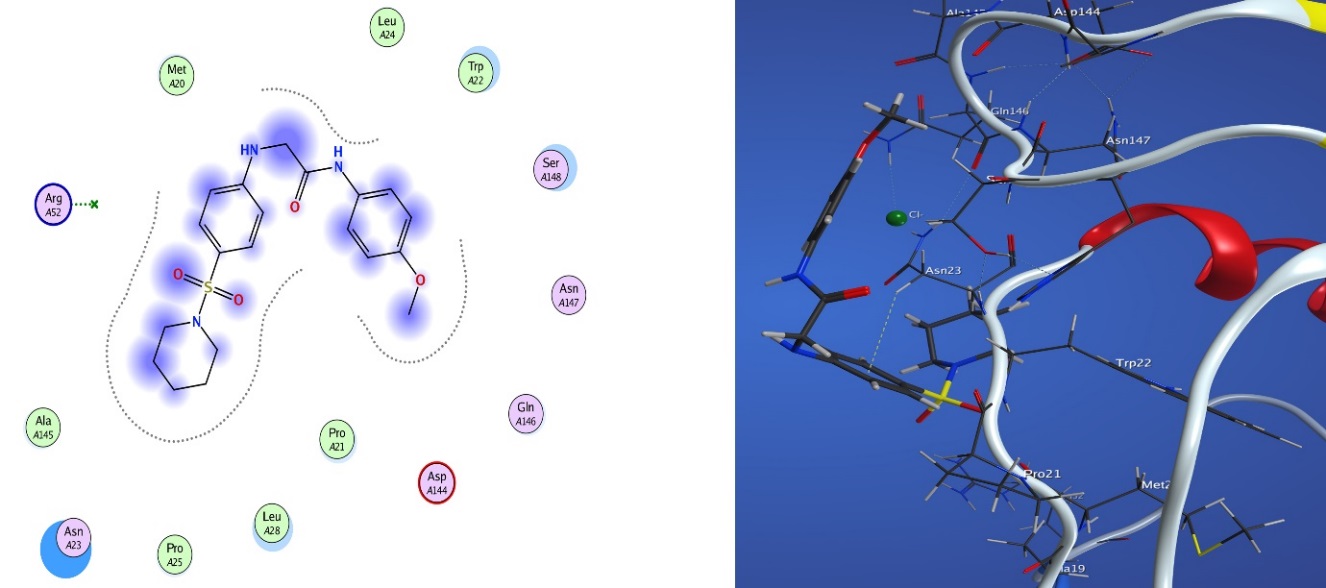
**

**Figure S16**. Docking of compound **6b** into DHFR

**Docking of compound 6c into DHFR**

The active site revealed that almost all atoms make hydrophobic interactions between many amino acid residues: Arg 52, Ala 19, Asp 144, Pro 21, Ala 145, Trp 22, Leu 28, Leu 24, Met 20, Ser 148, Asn 147, Asn 23, Gln 146, as shown in (**Figure S17**).

**
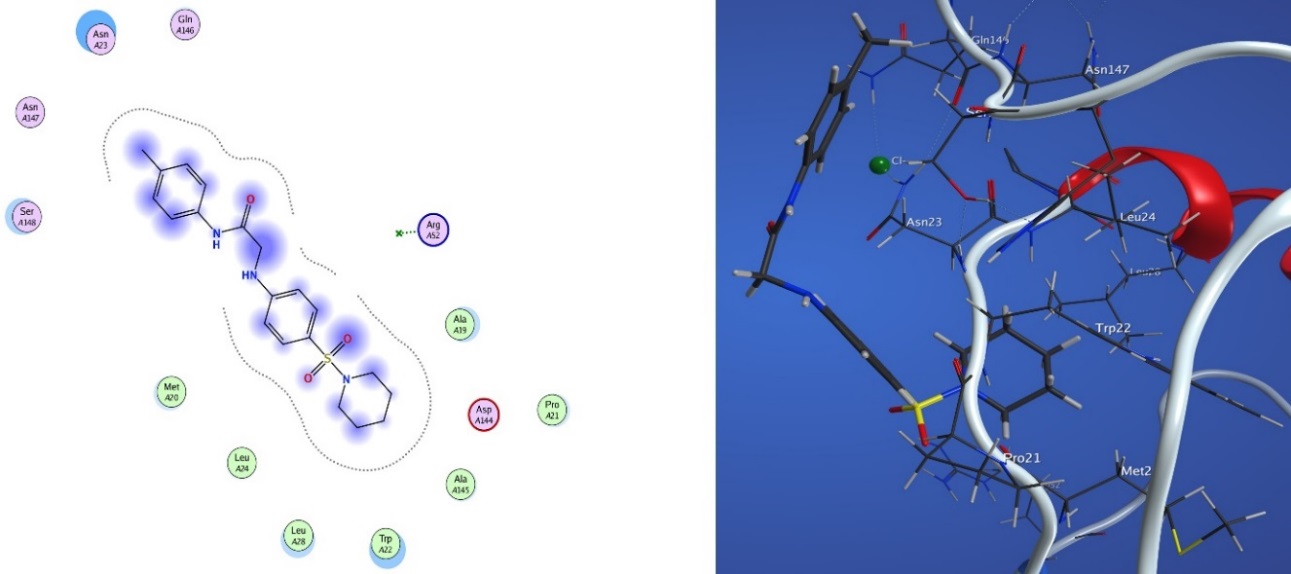
**

**Figure S17**. Docking of compound **6c** into DHFR

**Docking of compound 6e into DHFR**

The active site revealed that almost all atoms make hydrophobic interactions between many amino acid residues: Gln 146, Met 20, Asp 144, Trp 22, Pro 21, Ala 145, Ala 19, Arg 52, Asn 147, Asn 23, Ser 148, as shown in (**Figure S18**).

**
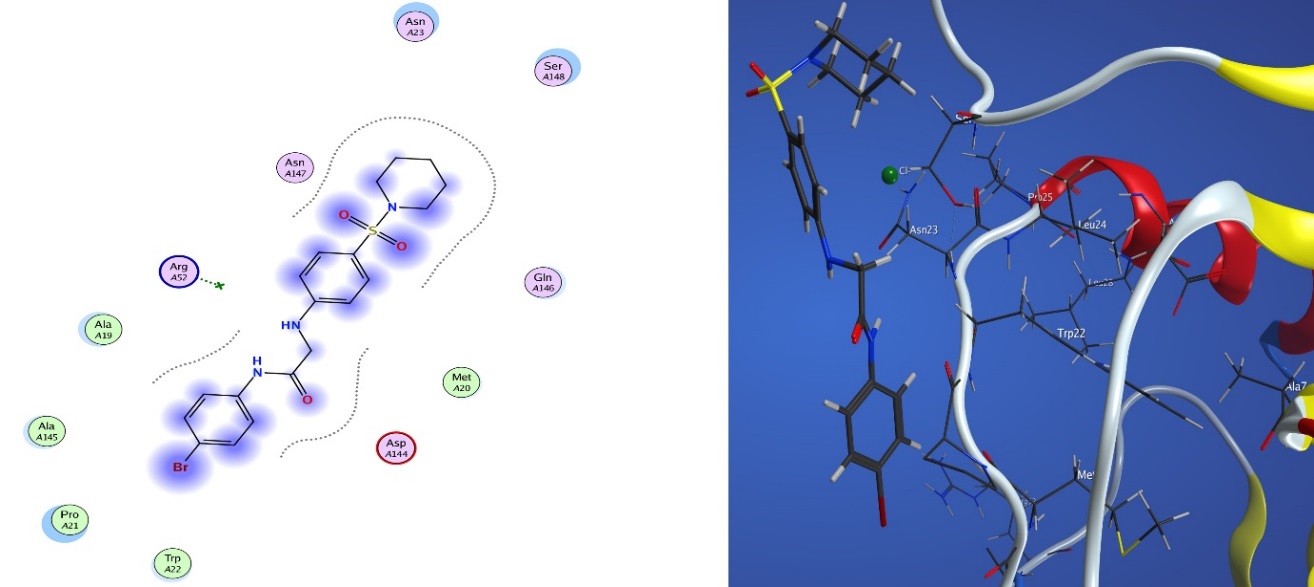
**

**Figure S18**. Docking of compound **6e** into DHFR

**Docking of compound 6f into DHFR**

The active site revealed that almost all atoms make hydrophobic interactions between many amino acid residues: Met 20, Leu 28, Asp 144, Ala 145, Pro 25, Trp 22, Leu 24, Asn 147, Pro 21, Ser 148, Asn 23, Gln 146, Arg 52, as shown in (**Figure S19**).

**
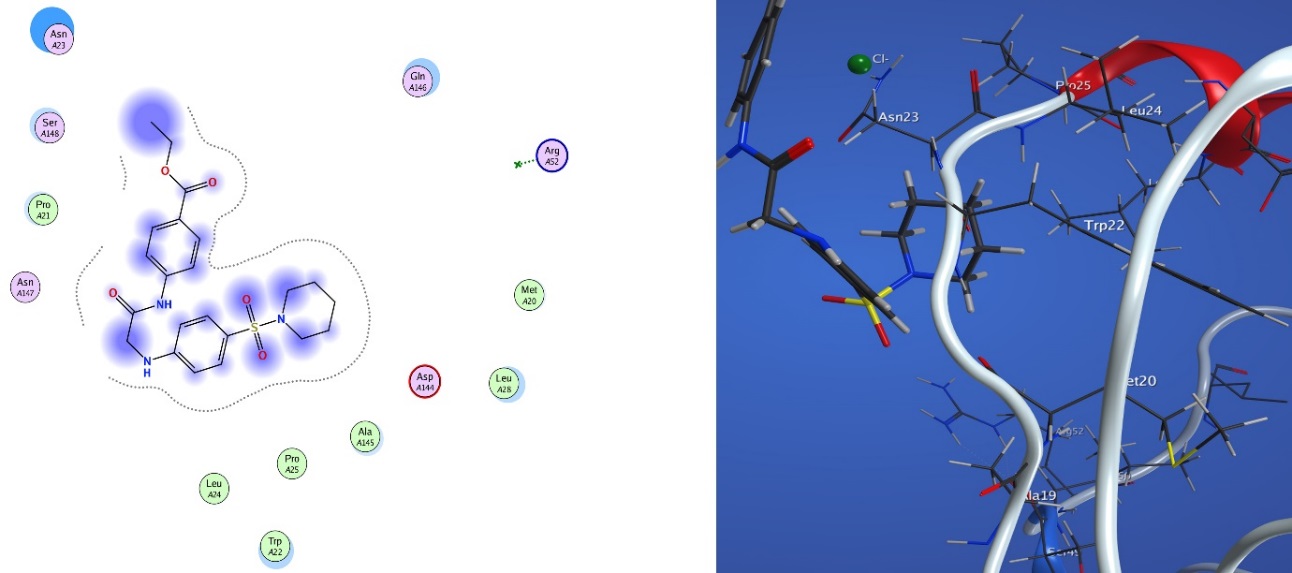
**

**Figure S19**. Docking of compound **6f** into DHFR

**Docking of compound 6g into DHFR**

The active site revealed the presence of ionic bond interactions between two oxygen atoms of carboxylic group and amino acid residue Arg 52 (3.96 Å and 3.66 Å) with energy -0.6 and -1.3 kcal/mol; respectively. Furthermore, it revealed the presence of an arene interaction between the phenyl ring and amino acid residue Ile 50 (4.54 Å) with energy -0.6 kcal/mol. This beside many hydrophobic interactions with various amino acid residues: Ala 19, Ser 49, Asn 23, Met 20, Leu 28, Trp 22, Leu 24, Pro 25, as shown in (**Figure S20**).

**
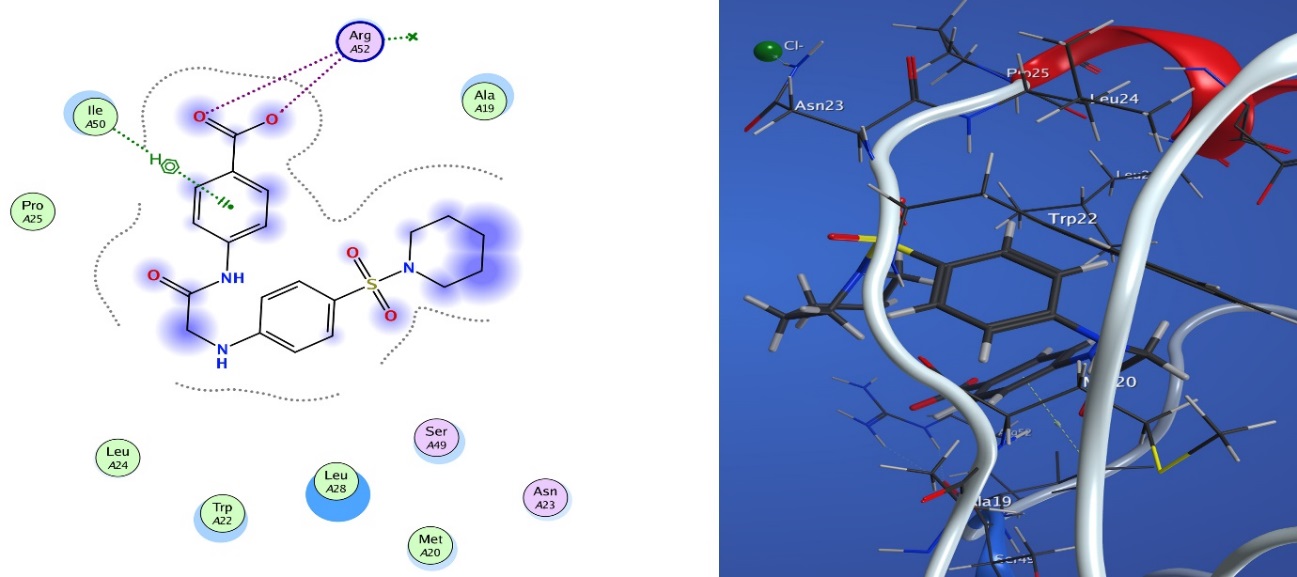
**

**Figure S20**. Docking of compound **6g** into DHFR

**Docking of compound 6i into DHFR**

The active site revealed the presence of one hydrogen bond interaction as nitrogen atom acted as a hydrogen bond donor with amino acid residue Trp 22 (2.96 Å) with energy -3.5 kcal/mol. Furthermore, it revealed the presence of an arene interaction between the phenyl ring and amino acid residue Asn 23 (3.59 Å) with energy -0.6 kcal/mol. This beside many hydrophobic interactions with various amino acid residues: Pro 25, Pro 21, Gln 146, Ser 148, Asn 147, Leu 24, Arg 52, Leu 28, as shown in (**Figure S21**).

**
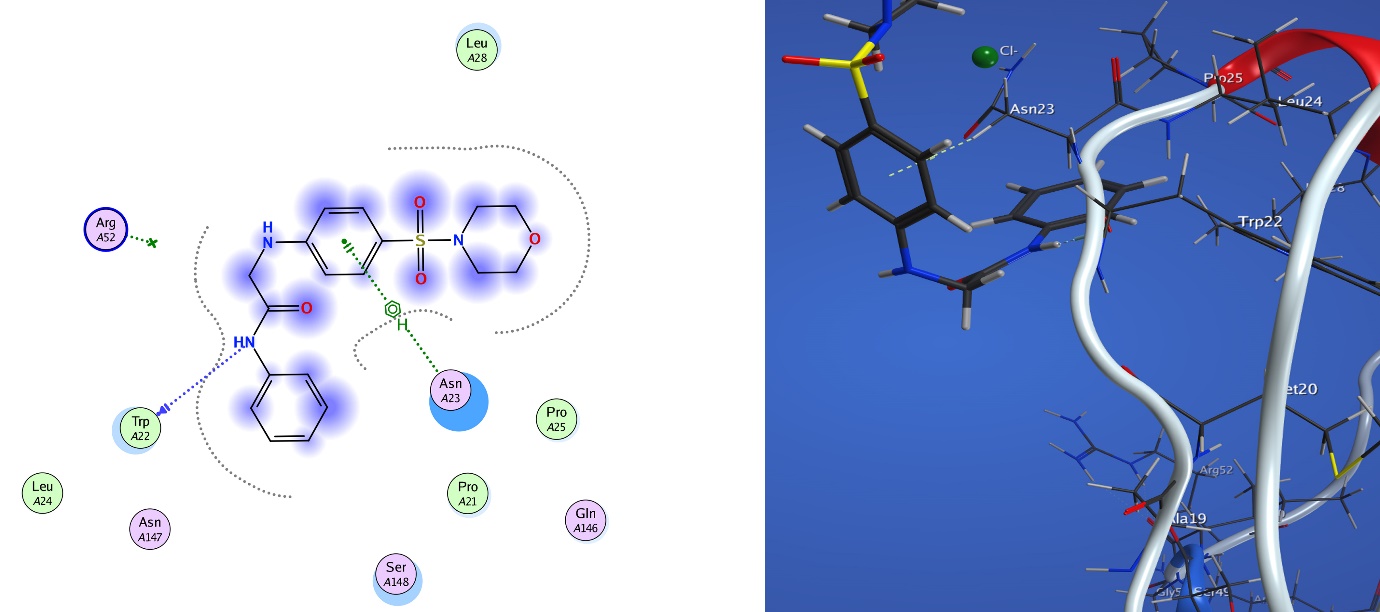
**

**Figure S21**. Docking of compound **6i** into DHFR

**Docking of compound 6j into DHFR**

The active site revealed the presence of two hydrogen bond interactions as nitrogen atom acted as a hydrogen bond donor with amino acid residues Ser 49 (3.10 Å) with energy -0.9 kcal/mol. While, oxygen atom of morpholine moiety acted as a hydrogen bond acceptor with amino acid residue Met 20 (3.43 Å) with energy -0.6 kcal/mol. This beside many hydrophobic interactions with various amino acid residues: Trp 22, Leu 28 Gly 51, Asn 23, Ile 50, Glu 48, Ala 19, Arg 52, as shown in (**Figure S22**).

**
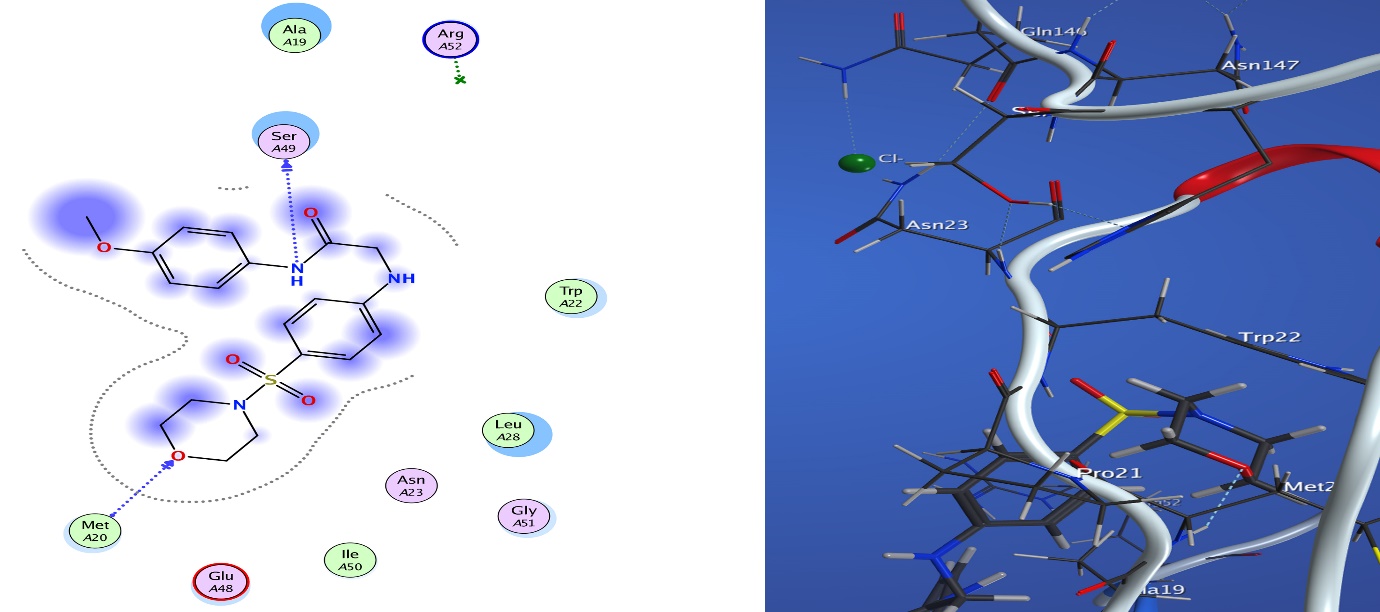
**

**Figure S22**. Docking of compound **6j** into DHFR

**Docking of compound 6k into DHFR**

The active site revealed that almost all atoms make hydrophobic interactions between many amino acid residues: Ser 148, Asn a47, Asn 23, Gln 146, Arg 52, Ala 145, Met 20, Trp 22, Asp 144, Pro 21, as shown in (**Figure S23**).

**
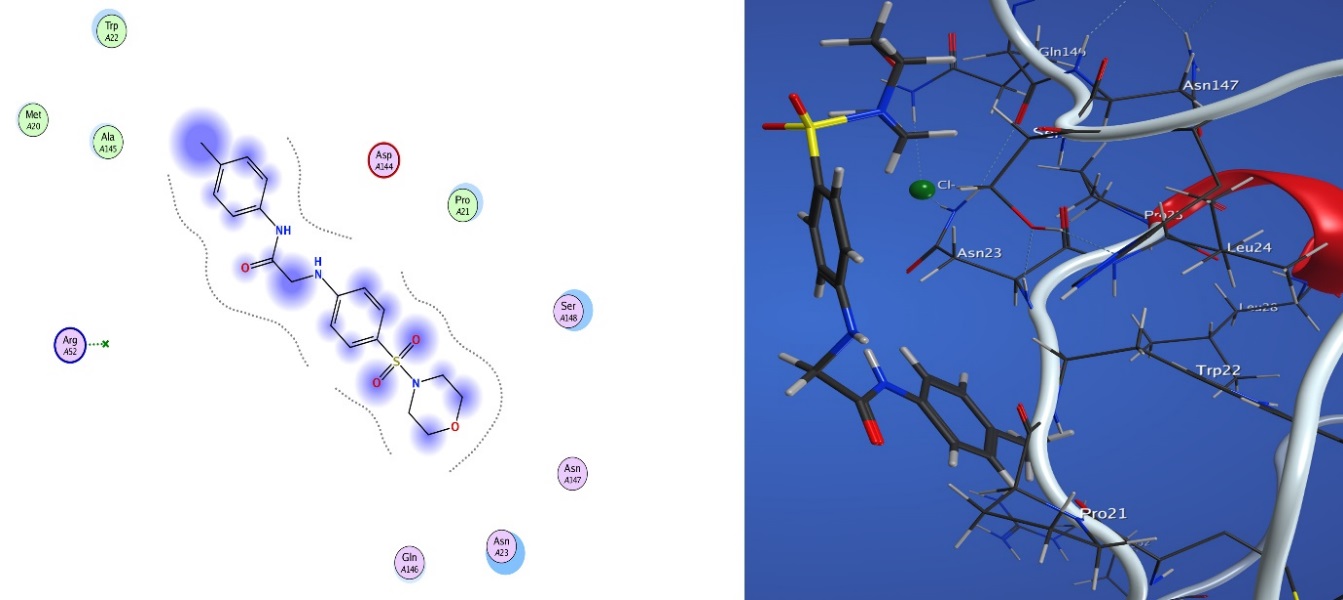
**

**Figure S23**. Docking of compound **6k** into DHFR

**Docking of compound 6l into DHFR**

The active site revealed that almost all atoms make hydrophobic interactions between many amino acid residues: Ser 148, Asn 147, Gln 146, Leu 24, Met 20, Arg 52, Asp 144, Ala 145, Leu 28, Pro 21, Trp 22, Asn 23, as shown in (**Figure S24**).

**
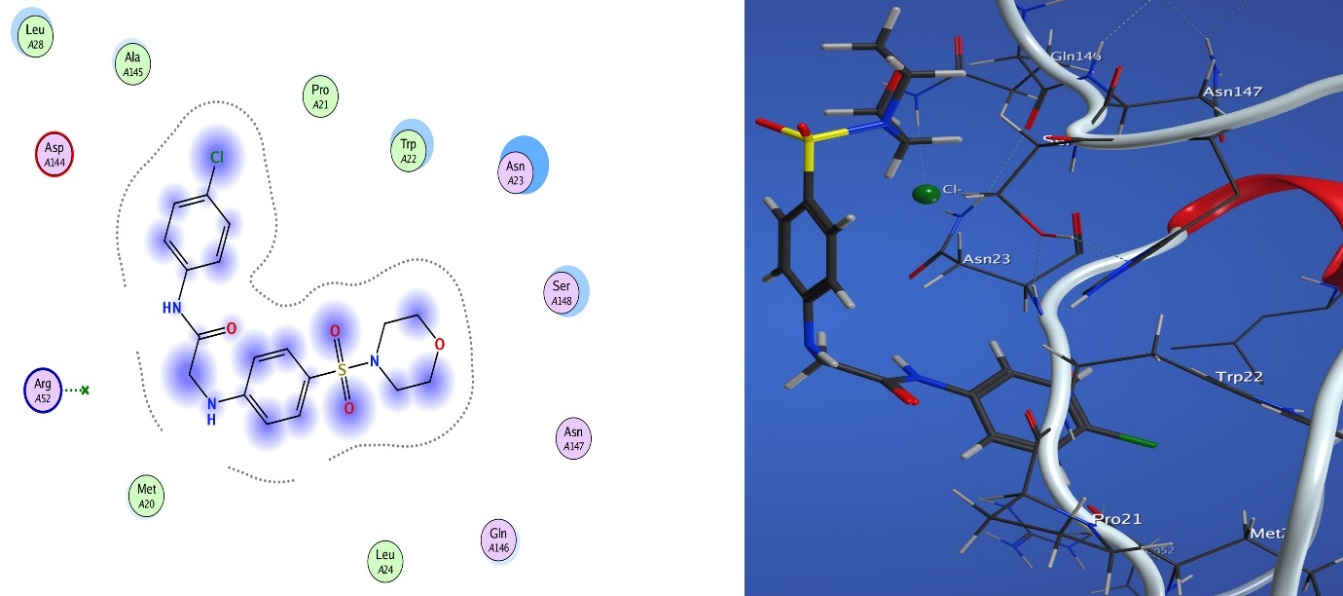
**

**Figure S24**. Docking of compound **6l** into DHFR

**Docking of compound 6m into DHFR**

The active site revealed that almost all atoms make hydrophobic interactions between many amino acid residues: Ser 148, Asn 147, Asn 23, Gln 146, Arg 52, Ala 19, Trp 22, Pro 21, Ala 145, Met 20, Asp 144, as shown in (**Figure S25**).

**
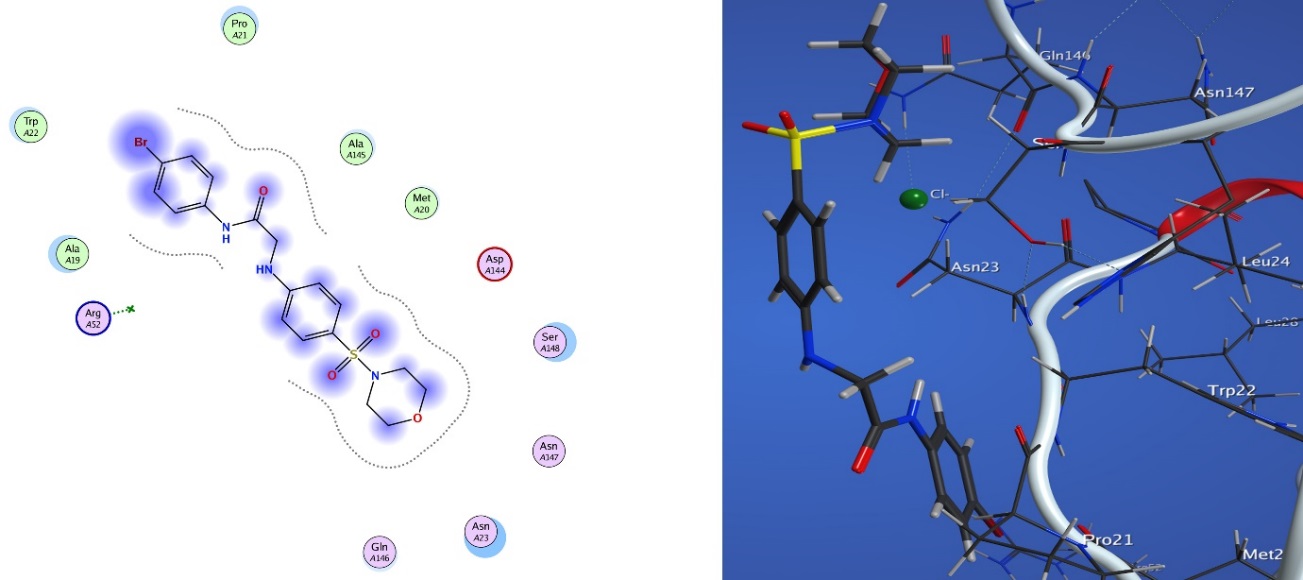
**

**Figure S25**. Docking of compound **6m** into DHFR

**Docking of compound 6n into DHFR**

The active site revealed the presence of one hydrogen bond interaction as one oxygen atom of SO2 moiety acted as a hydrogen bond acceptor with amino acid residue Leu 24 (3.10 Å) with energy -0.8 kcal/mol. This beside many hydrophobic interactions with various amino acid residues: Trp 22, Asn 23, Ser 49, Leu 28, Ile 50, Asp 27, Pro 25, Met 20, Arg 52, as shown in (**Figure S26**).

**
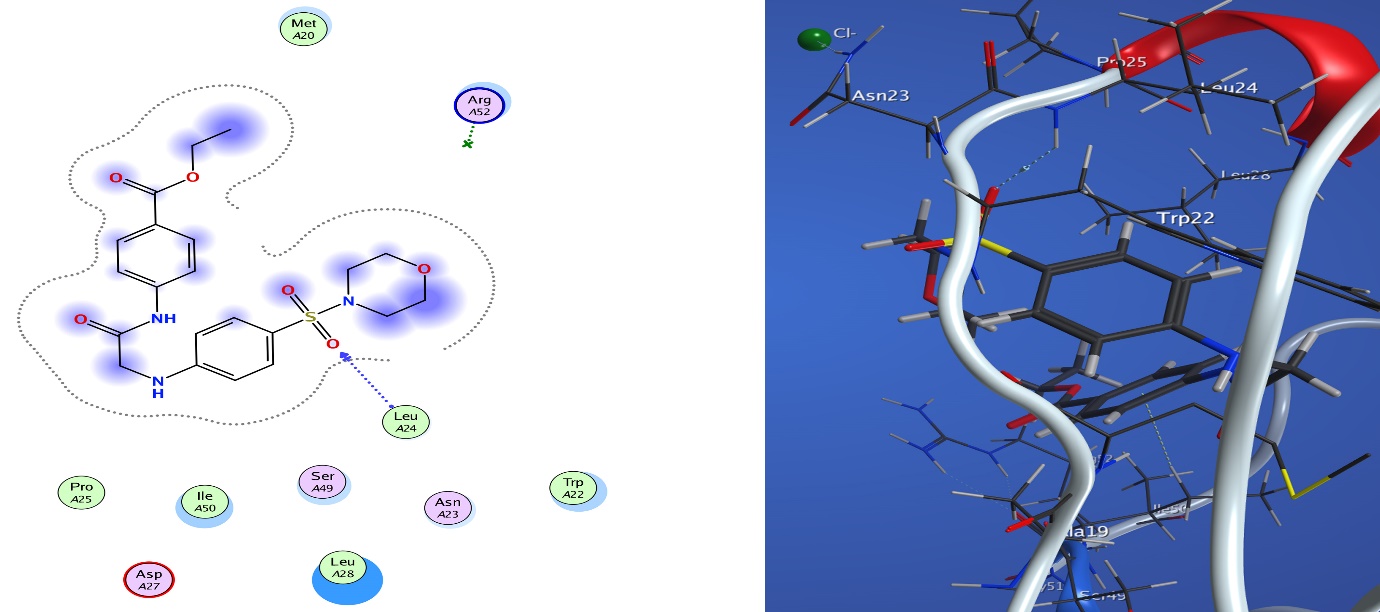
**

**Figure S26**. Docking of compound **6n** into DHFR

**Docking of compound 5o into DHFR**

The active site revealed the presence of one hydrogen bond interaction as oxygen atom of hydroxyl moiety acted as a hydrogen bond acceptor with amino acid residue Met 20 (3.11 Å) with energy -3.6 kcal/mol. This beside many hydrophobic interactions with various amino acid residues: Ile 50, Leu 24, Arg 52, Ala 19, Leu 28, Pro 25, Ser 49, Asn 23, Trp 22, as shown in (**Figure S27**).

**
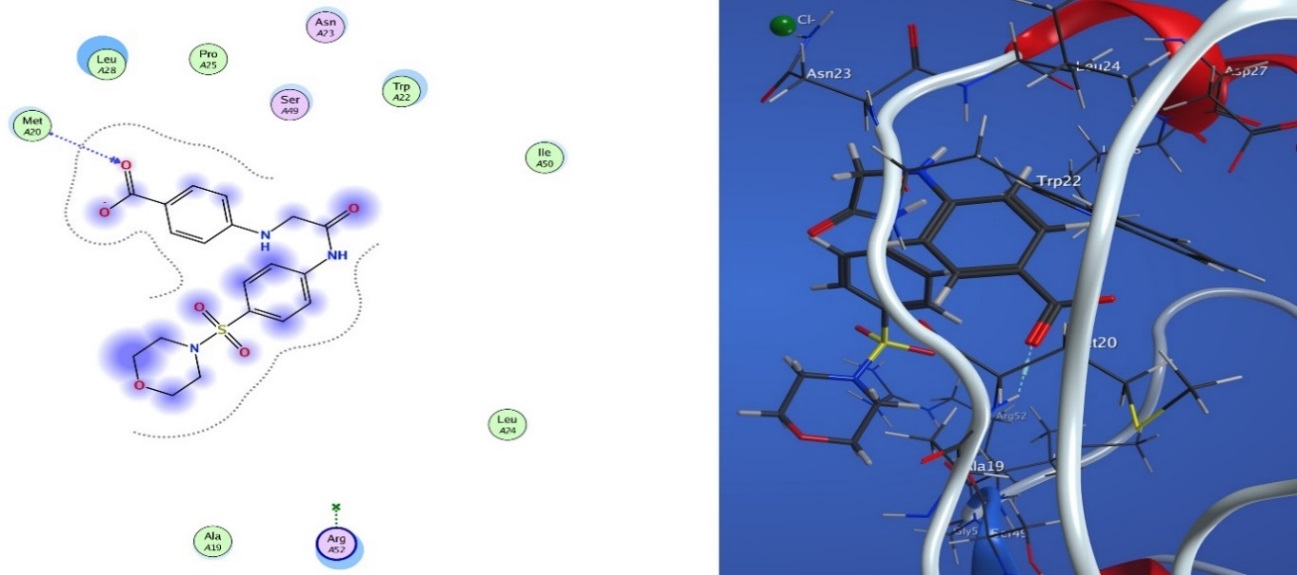
**

**Figure S27**. Docking of compound **5o** into DHFR

**Docking of compound 6p into DHFR**

The active site revealed the presence of one hydrogen bond interaction as one oxygen atom of nitro group acted as a hydrogen bond acceptor with amino acid residue Gln 146 (3.41 Å) with energy -0.7 kcal/mol. Furthermore, it revealed the presence of an arene interaction between the phenyl ring and amino acid residue Asn 23 (4.02 Å) with energy -0.9 kcal/mol. This beside many hydrophobic interactions with various amino acid residues: Arg 52, Trp 22, Ser 148, Ala 145, Leu 28, Leu 24, Pro 25, Pro 21, as shown in (**Figure S28**).

**
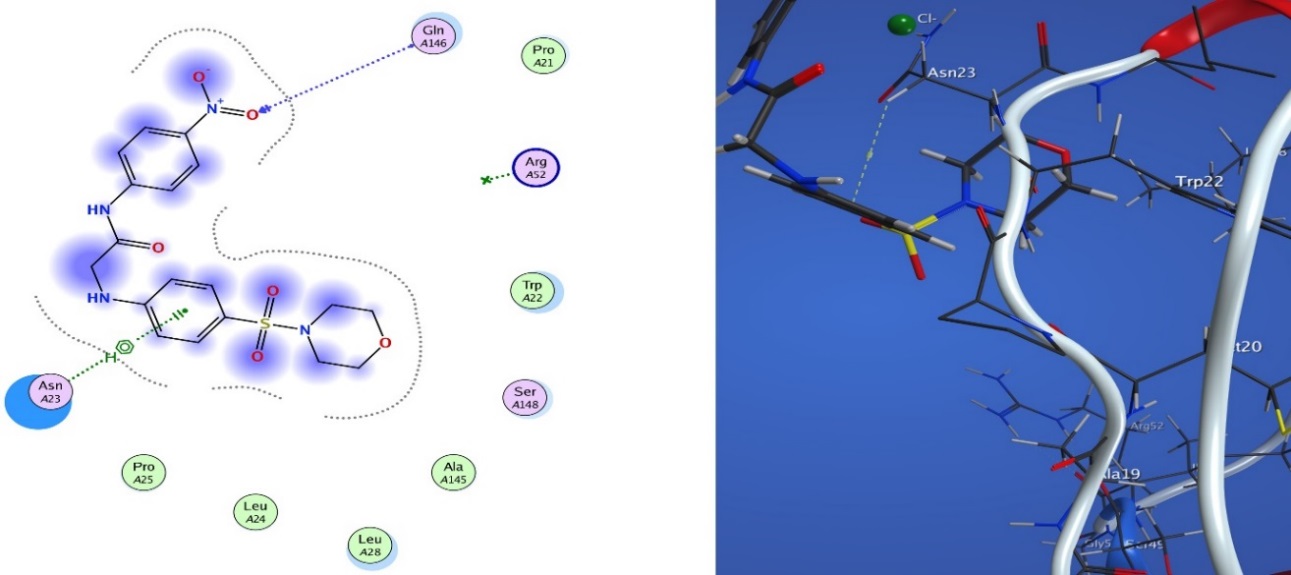
**

**Figure S28**. Docking of compound **6p** into DHFR
